# Performance of artificial intelligence models for monitoring psychological stress using noncontact physiological signals: a systematic review and meta-analysis

**DOI:** 10.3389/fpsyt.2026.1938767

**Published:** 2026-08-19

**Authors:** Xiuping Han, Qiankun Wang, Ling’ai Gao, Hui Cao

**Affiliations:** 1 Yangtze Delta Region Institute of Tsinghua University, Jiaxing, Zhejiang, China; 2 Linping District Integrated Traditional Chinese and Western Medicine Hospital, Hangzhou, Zhejiang, China; 3 Keeson Technology Corporation Limited, Jiaxing, Zhejiang, China

**Keywords:** artificial intelligence, digital mental health, noncontact monitoring, psychological stress, systematic review

## Abstract

**Background:**

Psychological stress is a common and dynamic mental health concern, but conventional assessment methods often rely on self-report questionnaires, clinical interviews, or contact-based physiological sensors, which may limit continuous and unobtrusive monitoring in everyday settings. Noncontact sensing technologies combined with artificial intelligence (AI) provide a potential approach for low-burden assessment by capturing physiological or physiology-related information without direct body contact.

**Objective:**

This systematic review and meta-analysis aimed to evaluate the performance of AI models using noncontact physiological signals for psychological stress recognition, summarize current sensing and modeling approaches, and identify methodological limitations affecting practical translation.

**Methods:**

We searched PubMed, IEEE Xplore, Web of Science, Embase, and APA PsycINFO from inception to May 6, 2026, and performed backward citation searching. Eligible studies used noncontact sensing modalities, including remote or imaging photoplethysmography, thermal imaging, or wireless radar, combined with machine learning or deep learning methods to identify psychological stress. Narrative synthesis was conducted for all included studies, and quantitative meta-analysis was performed for binary stress classification tasks.

**Results:**

Twenty-one studies were included, most of which were conducted in laboratory or controlled settings. For binary stress classification, 8 studies contributing 17 model-level estimates and 13,910 sample instances were included in the primary meta-analysis. The pooled accuracy was 81.3% (95% CI 68.4%–91.5%), with substantial heterogeneity. Supplementary analyses showed pooled F1 score, sensitivity, and specificity values of 0.791, 0.809, and 0.697, respectively. Exploratory subgroup analyses suggested that stress label sources were associated with model performance, whereas sensing modality, signal category, validation approach, and model type showed no statistically significant differences.

**Conclusions:**

Current evidence supports the technical feasibility of noncontact AI-based approaches for psychological stress recognition. However, available studies remain limited by small samples, heterogeneous stress labels, insufficient participant-independent validation, incomplete performance reporting, and limited evaluation in real-world settings. Future research should prioritize standardized stress labeling, transparent model validation, comprehensive reporting of performance metrics, and naturalistic evaluation to improve the reliability and applicability of noncontact AI-based mental health monitoring approaches.

## 1 Introduction

Psychological stress is an important public health concern and a key issue in digital mental health management. Global survey data indicate that approximately 37% of adults worldwide reported experiencing high levels of stress on the day preceding the survey in 2024 (1). Psychological stress generally refers to the psychological and physiological responses that arise when individuals perceive environmental demands as exceeding their available coping resources (2, 3). As a multidimensional process involving cognitive appraisal, emotional responses, behavioral coping, and autonomic regulation, psychological stress can affect emotional well-being, cognitive functioning, sleep quality, and social functioning (4–7). Prolonged or repeated exposure to stress has also been associated with an increased risk of anxiety, depression, sleep disorders, and cardiovascular conditions (8–10). Therefore, timely and objective identification of psychological stress is important for mental health risk screening, early intervention, and digital health management.

Current approaches to psychological stress assessment can be broadly categorized as subjective or objective. Subjective assessment commonly relies on self-report questionnaires, standardized scales, or clinical interviews, such as the 10-item Perceived Stress Scale (PSS-10) (11) and the State-Trait Anxiety Inventory (STAI) (12). These methods can capture individuals’ cognitive appraisals and emotional experiences, but they are susceptible to recall bias, social desirability bias, individual differences in self-expression, current emotional states, and assessment context. They also typically provide retrospective assessments over a specified period and are therefore limited in capturing dynamic changes in stress under real-world conditions. Objective assessments aim to capture stress-related responses through behavioral, physiological, or biochemical indicators, including heart rate, heart rate variability, blood volume pulse, skin temperature, respiration, electrodermal activity, cortisol, facial expressions, blinking, and pupillary changes (13, 14). Physiological signals are particularly relevant because they can reflect dynamic changes in autonomic nervous system activity and stress-response processes and are suitable for continuous measurement and computational modeling.

Contact-based and wearable physiological monitoring represents one of the most commonly used technical approaches in AI-based stress recognition. Previous studies have collected physiological data using electrocardiography (ECG), electrodermal activity (EDA), photoplethysmography (PPG), respiratory belts, or wearable devices and subsequently applied machine learning or deep learning methods to identify stress states (15, 16). These approaches have provided important evidence supporting automated psychological stress recognition. However, their real-world application may be affected by wearing discomfort, variations in skin contact, motion artifacts, and poor adherence during long-term monitoring (15, 16). In contrast, noncontact monitoring technologies can acquire stress-related physiological information without direct physical contact, providing an alternative approach to continuous and unobtrusive psychological stress monitoring.

Noncontact stress monitoring typically extracts stress-related physiological or physiology-related features using cameras, infrared or thermal imaging devices, and wireless sensing technologies (13). Remote or imaging photoplethysmography (rPPG/iPPG) can estimate heart rate, pulse-to-pulse intervals, and heart rate variability from facial videos (17). Thermal imaging can capture the spatial distribution and dynamic changes of facial skin temperature, thereby reflecting peripheral temperature variations associated with autonomic nervous system activity (18). Wireless radar and other wireless sensing technologies can acquire information related to respiration, body movement, and cardiopulmonary activity without physical contact (19). These approaches offer potential advantages in terms of low user burden, minimal interference, and ease of integration into naturalistic environments.

Although a growing body of research has investigated noncontact stress monitoring, substantial heterogeneity exists across studies in sensing modalities, sources of stress labels, classification tasks, algorithmic models, and performance metrics, limiting direct comparisons between findings. Reported model performance may also be influenced by sample size, data partitioning procedures, participant-level independence between training and test sets, and validation strategies. Consequently, the high classification performance reported in some individual studies should be interpreted with caution, and the overall performance and practical utility of noncontact AI-based stress monitoring remain to be systematically evaluated.

Accordingly, this systematic review and meta-analysis aimed to evaluate the performance of AI models using noncontact physiological signals for psychological stress monitoring, summarize current sensing approaches and modeling strategies, and identify methodological limitations affecting clinical translation.

## 2 Materials and methods

### 2.1 Overview

This systematic review and meta-analysis was conducted and reported in accordance with the Preferred Reporting Items for Systematic Reviews and Meta-Analyses for Diagnostic Test Accuracy Studies (PRISMA-DTA) statement (20). The completed PRISMA-DTA checklist is provided in **Supplementary Material 1**. This review was not prospectively registered.

### 2.2 Search strategy

To identify eligible studies, a comprehensive search of five electronic databases was conducted on May 6, 2026, including PubMed, IEEE Xplore, Web of Science, Embase, and APA PsycINFO. In addition, to minimize the risk of missing relevant studies, backward citation searching was performed by manually screening the reference lists of relevant articles and included studies.

The search strategy was developed through consultation with two experts in digital mental health and by reviewing relevant systematic reviews and original studies. It was structured around five concept blocks: psychological stress, detection tasks, noncontact or unobtrusive sensing approaches, physiological or physiology-related signals, and artificial intelligence or machine learning methods. The psychological stress block included terms related to stress, mental stress, psychological stress, perceived stress, emotional stress, acute stress, cognitive stress, psychosocial stress, stress level, and stress state. The detection task block included terms related to detection, recognition, classification, prediction, monitoring, identification, assessment, estimation, and discrimination. The noncontact or unobtrusive sensing block included terms related to contactless or unobtrusive monitoring, camera-based and video-based sensing, thermal imaging, radar-based and wireless sensing, remote photoplethysmography, imaging photoplethysmography, and bed- or chair-based sensing approaches. The physiological or physiology-related signal block included terms related to vital signs, autonomic and cardiorespiratory signals, photoplethysmography, heart rate, heart rate variability, respiration, skin temperature, body temperature, oxygen saturation, and pulse wave characteristics. The artificial intelligence and machine learning block included terms related to artificial intelligence, machine learning, deep learning, supervised learning, classification models, and commonly used algorithms, including support vector machines, random forests, decision trees, k-nearest neighbors, neural networks, boosting algorithms, logistic regression, naive Bayes, and linear discriminant analysis.

Within each concept block, search terms were combined using the Boolean operator “OR”, whereas the five concept blocks were combined using the Boolean operator “AND”. To improve the specificity of the search and reduce irrelevant records, terms related to non-psychological forms of stress, such as oxidative stress, heat stress, thermal stress, mechanical stress, cell stress, plant stress, crop stress, drought stress, salt stress, water stress, and stress-strain, were excluded. In addition, non-original research articles, including reviews, systematic reviews, meta-analyses, survey papers, and protocols, were excluded through the search strategy where applicable. Because search syntax, field tags, truncation rules, and controlled vocabulary differ across databases, the search strategy was adapted to the requirements of each database. The complete search strategies are provided in Multimedia Appendix 2.

### 2.3 Eligibility criteria

Studies were eligible for inclusion if they met all of the following criteria: (i) the participants were human, with no restrictions on sex, age, occupation, country, or geographic region; (ii) the study focused on the detection, monitoring, or classification of psychological stress, acute stress, academic stress, occupational stress, driving-related stress, or other psychosocial stress states; (iii) noncontact physiological signals were used as the primary data source; (iv) artificial intelligence, machine learning, or deep learning methods were used to develop a model for psychological stress recognition; (v) at least one extractable model performance metric was reported, such as accuracy, F1 score, area under the receiver operating characteristic curve (AUC), sensitivity, specificity, precision, or recall; and (vi) the study was an original empirical investigation published as a journal article, full-length conference paper, or preprint. Eligible study settings included laboratory-based stress induction experiments, simulated scenarios, and real-world or semi-naturalistic settings such as classrooms, workplaces, and driving environments.

Studies were excluded if they met any of the following criteria: (i) they were reviews, systematic reviews, meta-analyses, conference abstracts, study protocols, commentaries, editorials, book chapters, or other nonoriginal publications; (ii) the primary objective was not the recognition of psychological stress; (iii) only contact-based or wearable physiological signals, or physiological or behavioral features derived from such devices, were used as model inputs, and results for models based solely on noncontact signals could not be extracted separately; (iv) no model performance metrics suitable for data extraction were reported, or the full text did not provide sufficient information; or (v) the study conducted a secondary analysis of a publicly available dataset or a dataset from a previous study without collecting new, original noncontact signal data.

For studies that reported both multimodal fusion models and models based on a single noncontact physiological modality, we preferentially extracted results from models that met the definition of noncontact physiological monitoring used in this review. If the relevant results could not be separated from those based on contact-based signals, wearable signals, or purely behavioral features, they were excluded from the quantitative synthesis.

### 2.4 Study selection

The study selection process consisted of three stages: deduplication, title and abstract screening, and full-text assessment. First, records retrieved from all databases were imported into EndNote 2025 for initial duplicate removal. The remaining records were then uploaded to the Rayyan online platform, where additional manual deduplication was performed based on information such as title, author names, publication year, journal name, and DOI. Subsequently, two reviewers independently conducted title and abstract screening in Rayyan according to the predefined eligibility criteria, and records that clearly did not meet the inclusion criteria were excluded. Records for which eligibility could not be determined at this stage were retained for full-text assessment. Finally, the full texts of potentially eligible studies were independently reviewed by two reviewers, and the final studies included in the systematic review were determined according to the prespecified inclusion and exclusion criteria. Any uncertainties or disagreements during the screening process were resolved through discussion; if consensus could not be reached, a third reviewer was consulted for adjudication.

### 2.5 Data extraction and statistical analysis

The data extraction form was pilot-tested using 3 studies and subsequently refined (Multimedia Appendix 3). Two reviewers independently used Microsoft Excel to extract information on study characteristics, participant characteristics, types of noncontact signals, device characteristics, sources of stress labels, model input features, AI algorithms, validation methods, and model performance metrics. Disagreements were resolved through discussion. For studies reporting raw data or confusion matrices, stress was consistently treated as the positive class, and performance metrics, including accuracy, sensitivity, specificity, and F1 score, were calculated or recalculated when necessary. As some studies reported multiple models based on different algorithms, sensing modalities, feature combinations, or validation settings, all eligible results were extracted as model-level effect estimates.

The findings were synthesized using a combination of narrative and quantitative approaches. Narrative synthesis was used to summarize the characteristics of the included studies, technical approaches, model development procedures, and reporting of model performance. Quantitative synthesis was restricted to studies involving binary stress classification, with accuracy specified as the primary pooled outcome. F1 score, sensitivity, and specificity were treated as supplementary outcomes and were synthesized exploratively when sufficient data were available. Diagnostic odds ratios and likelihood ratios were also reported as supplementary analyses.

Because individual studies could contribute multiple correlated effect estimates, multilevel random-effects models were used for the meta-analyses. Proportion-based outcomes, including accuracy, sensitivity, and specificity, were transformed using the Freeman–Tukey double-arcsine transformation before model fitting and back-transformed after pooling. F1 scores were synthesized after logit transformation. Heterogeneity was assessed using variance components from the multilevel models, including between-study variance and within-study or effect estimate–level variance.

To explore potential sources of between-study variation, exploratory subgroup analyses were conducted to examine the associations of sensing modality, signal category, source of stress labels, validation approach, and model type with accuracy. Differences between subgroups were tested using multilevel meta-regression models. Given the limited number of included studies, small-sample inference based on t and F distributions was used to reduce the risk of false-positive findings. All statistical analyses were performed using R software.

### 2.6 Risk of bias and applicability assessment

To assess the methodological quality of the included studies, we adapted the Quality Assessment of Diagnostic Accuracy Studies–2 (QUADAS-2) tool (21) to align with the objectives of this review, drawing on the quality and risk-of-bias assessment approach used in a previous related review (22). The adapted QUADAS-2 tool comprised 4 domains: participant selection, index test, reference standard, and analysis. Four signaling questions were developed within each domain to reflect the specific objectives and methodological considerations of this review.

Two reviewers independently assessed all included studies using the adapted QUADAS-2 tool provided in Multimedia Appendix 4. Any disagreements between the reviewers were resolved through discussion and consensus.

## 3 Results

### 3.1 Search results

**Figure 1:**
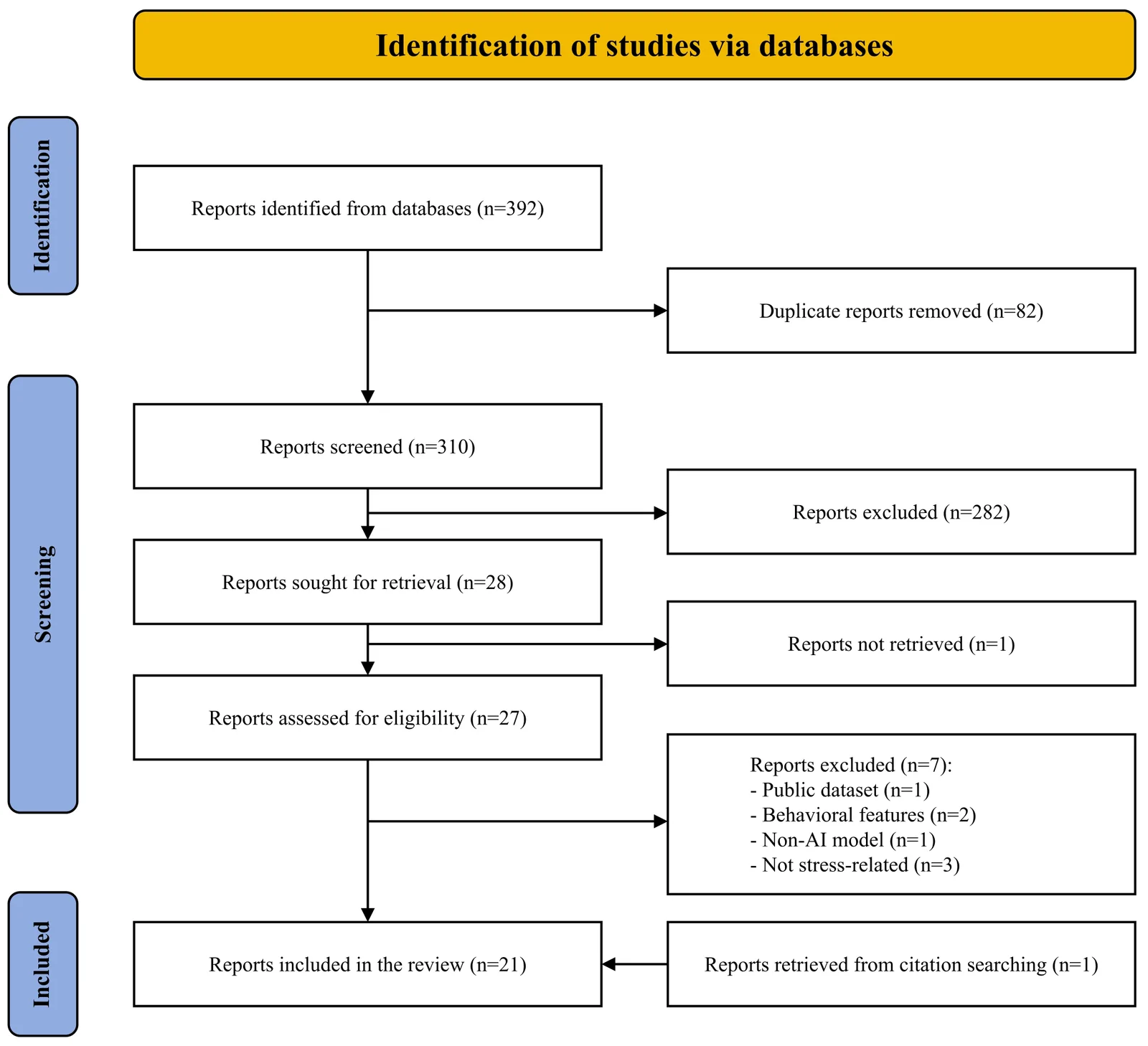
Flowchart of the study selection process.

As shown in **Figure 1**, the database searches identified 392 records. After 82 duplicates were removed using EndNote 2025, 310 records remained for title and abstract screening. Of these, 282 records that did not meet the eligibility criteria were excluded, leaving 28 reports for full-text retrieval and eligibility assessment. The full texts of 27 reports were successfully retrieved, whereas 1 report could not be obtained.

Following full-text assessment, 7 reports were excluded. The reasons for exclusion were secondary analysis of a publicly available dataset (n=1), use of behavioral features only (n=2), use of a non-AI model (n=1), and a study objective unrelated to psychological stress recognition (n=3). Backward citation searching identified 1 additional eligible study.

Ultimately, 21 studies were included in the systematic review, of which 8 were included in the primary meta-analysis.

### 3.2 Characteristics of the included studies

The main characteristics of the included studies are presented in **Table 1**. A total of 21 studies published between 2014 and 2026 were included in this review. The largest number of studies was published in 2022 (4/21, 19.0%), followed by 2024 (3/21, 14.3%). Most were journal articles (16/21, 76.2%), while the remainder were conference papers (4/21, 19.0%) and a preprint (1/21, 4.8%).

**Table 1:** Characteristics of the included studies (N = 21).

Characteristic	Value	References
Publication year, n (%)		
2026	1 (4.8)	(23)
2025	2 (9.5)	(18, 24)
2024	3 (14.3)	(17, 25, 26)
2022	4 (19.0)	(27–30)
2021	2 (9.5)	(19, 31)
2020	1 (4.8)	(32)
2019	1 (4.8)	(33)
2018	2 (9.5)	(34, 35)
2017	1 (4.8)	(36)
2016	1 (4.8)	(37)
2015	1 (4.8)	(38)
2014	2 (9.5)	(39, 40)
Publication type, n (%)		
Journal article	16 (76.2)	(17–19, 23–29, 31, 33–35, 37, 40)
Conference paper	4 (19.0)	(30, 32, 38, 39)
Preprint	1 (4.8)	(36)
Country of participants, n (%)		
China	3 (14.3)	(25, 29, 31)
Mexico	2 (9.5)	(23, 27)
France	2 (9.5)	(26, 37)
Russia	3 (14.3)	(32, 34, 35)
USA	2 (9.5)	(19, 38)
UK	2 (9.5)	(33, 36)
Poland	1 (4.8)	(18)
Portugal	1 (4.8)	(17)
India	1 (4.8)	(24)
Italy	1 (4.8)	(28)
Iran	1 (4.8)	(39)
Australia	1 (4.8)	(40)
Germany	1 (4.8)	(30)
Number of participants, mean (SD; range)	42.05 (32.47;8-120)	(17–19, 23–40)
Age (y)		
Mean (SD), n (%)	23.29 ± 5.63 (6,28.6)	(25, 28, 30, 33, 35, 36)
Range, n (%)	18-50 (12,57.1)	(17–19, 26, 27, 29, 31, 32, 34, 37, 38, 40)
NR^a^, (n,%)	3 (14.3)	(23, 24, 39)
Female participants		
Values (%)	40.42	(17, 19, 23, 25–28, 32–34, 36, 37, 40)
NR^a^, n (%)	7 (33.3)	(18, 23, 24, 29–31, 38, 39)
Educational level, n (%)		
Below high school	1 (4.8)	(17)
High school	1 (4.8)	(17)
Undergraduate	6 (28.6)	(17, 23, 27, 32, 34, 35)
Postgraduate	2 (9.5)	(31, 40)
NR^a^, n (%)	13 (61.9)	(19, 24–26, 28–30, 33, 36–39)

^a^ NR: not reported.

The included studies were conducted in 13 countries, with China and Russia contributing the largest numbers of studies (3/21, 14.3% each). Sample sizes ranged from 8 to 120 participants, with a mean sample size of 42.05 (SD 32.47). Participant age was incompletely reported. Only 6 studies reported both the mean and SD of age (6/21, 28.6%), with an average participant age of 23.29 years (SD 5.63). An additional 12 studies reported age ranges only (12/21, 57.1%), with an overall range of 18 to 50 years.

The proportion of female participants was reported in 13 studies (13/21, 61.9%), with a mean proportion of 40.42%. Reporting of educational level was limited, and most studies did not provide information on participants’ educational backgrounds (13/21, 61.9%). Among studies that reported educational information, undergraduate students were the most common participant group (6/21, 28.6%). Detailed characteristics of the included studies are provided in **Table 1**.

### 3.3 Characteristics of noncontact devices

As shown in **Table 2**, all included studies used noncontact data acquisition methods (21/21, 100%). Cameras were the most commonly used acquisition devices, accounting for 52.4% of the studies (11/21), followed by thermal imaging devices (8/21, 38.1%) and wireless radar–based devices (4/21, 19.0%). With regard to the body region monitored, the face was the primary site of signal acquisition (16/21, 76.2%). The thoracic and abdominal regions were monitored in 4 studies (4/21, 19.0%), mainly for the acquisition of radar-based or respiration-related noncontact signals. One study collected signals from both the face and hands (1/21, 4.8%). Most studies were conducted in controlled laboratory environments (19/21, 90.5%). Semi-naturalistic settings were used in 1 study (1/21, 4.8%), while naturalistic environments or everyday application settings were investigated in 3 studies (3/21, 14.3%).

**Table 2:** Characteristics of the noncontact devices used in the included studies (N = 21).

Characteristic	Value	References
Type of acquisition device, n (%)		
Wireless radar	4 (19.0)	(19, 32, 34, 35)
Camera	11 (52.4)	(17, 24–31, 37, 38, 40)
Thermal imaging device	8(38.1)	(18, 23, 25, 28, 33, 36, 39, 40)
Body region monitored, n (%)		
Facial region	16 (76.2)	(17, 24–31, 33, 36–40)
Face and hands	1 (4.8)	(23)
Thoracic and abdominal regions	4 (19.0)	(19, 32, 34, 35)
Data collection setting, n (%)		
Controlled laboratory setting	19 (90.5)	(18, 19, 23, 25–40)
Semi-naturalistic setting	1 (4.8)	(24)
Naturalistic setting	3 (14.3)	(17, 19, 29)
Use of fully noncontact data acquisition	21 (100)	(17–19, 23–40)

Because some studies used multiple acquisition devices, monitored more than one body region, or included multiple study settings, the totals across categories may exceed the total number of included studies.

### 3.4 Features of the AI

As shown in **Table 3**, all included studies used machine learning or deep learning methods to develop stress classification models. Binary classification was the predominant task (17/21, 81.0%). Traditional machine learning algorithms were used most frequently, particularly SVMs (12/21, 57.1%), random forests (7/21, 33.3%), and k-nearest neighbors (6/21, 28.6%). Convolutional neural networks (CNNs) and other neural network models were used less frequently. Regarding model inputs, studies primarily used heart rate and related features (13/21, 61.9%), skin temperature–related features (7/21, 33.3%), and respiration-related information (5/21, 23.8%). A small number of studies additionally incorporated facial expressions or behavioral features or used iPPG signals directly as model inputs. Arithmetic and related cognitive tasks were the most commonly used methods of stress induction (12/21, 57.1%), followed by Stroop tasks (4/21, 19.0%) and film- or image-based stimuli (3/21, 14.3%). K-fold cross-validation was the most commonly used model validation method (11/21, 52.4%), followed by leave-one-out cross-validation (8/21, 38.1%) and training-test set splits (7/21, 33.3%). All studies reported accuracy (21/21, 100.0%), whereas F1 score, sensitivity, specificity, and AUC were reported less frequently.

**Table 3:** Characteristics of AI models in the included studies (N = 21).

Feature	Values	References
Problem-solving approaches, n (%)		
Classification	21 (100)	(17–19, 23–40)
Number of stress classes, n (%)		
2	17 (81.0)	(17, 19, 25–28, 30–40)
3	4 (19.1)	(19, 24, 29, 36)
>3	2 (9.5)	(18, 23)
AI algorithms, n (%)		
SVM	11 (52.4)	(23, 25–29, 31, 35, 37, 39, 40)
Random forest	7 (33.3)	(17, 19, 25–27, 29, 30)
K-nearest neighbor	6 (28.6)	(23, 27, 29, 30, 33, 36)
CNN-based neural network	5 (23.8)	(18, 23, 24, 30, 36)
Decision tree	5 (23.8)	(25–27, 30, 32)
Feed-forward neural network	5 (23.8)	(29, 30, 33, 34, 36)
Boosting algorithms	3 (14.3)	(17, 30, 32)
Linear discriminant analysis	3 (14.3)	(37–39)
Extra Trees	2 (9.5)	(17, 32)
Logistic regression	2 (9.5)	(26, 38)
Naïve Bayes	2 (9.5)	(26, 30)
Hybrid neural network	1 (4.8)	(30)
Gaussian process	1 (4.8)	(30)
Data types, n (%)		
Heart rate and related features	13 (61.9)	(17, 19, 25–27, 29–33, 35, 37, 38)
Skin temperature and related features	7 (33.3)	(18, 23, 25, 28, 33, 39, 40)
Facial expression and behavioral features	4 (19.1)	(17, 24, 31, 40)
Imaging photoplethysmography signals	2 (9.5)	(24, 30)
Respiration-related information	5 (23.8)	(19, 32, 34–36)
Body movement–related information	1 (4.8)	(19)
Stress-induction methods, n (%)		
Arithmetic and related cognitive tasks	12 (57.1)	(18, 19, 25–27, 29, 31–36)
Stroop task	4 (19.1)	(28, 33, 36, 37)
Film- or image-based stimuli	3 (14.3)	(18, 38, 40)
No additional stress induction	2 (9.5)	(17, 29)
Trier Social Stress Test	1 (4.8)	(23)
Simulated driving task	1 (4.8)	(24)
Deception-detection paradigm	1 (4.8)	(39)
Sustained attention task	1 (4.8)	(30)
Types of validation, n (%)		
Training–test set split	7 (33.3)	(17–19, 23, 29, 31, 37)
K-fold cross-validation	11 (52.4)	(23, 24, 26–29, 32, 35, 37, 39, 40)
Leave-one-out cross-validation	8 (38.1)	(19, 28, 30, 31, 33, 34, 36, 38)
Nested cross-validation	2 (9.5)	(17, 29)
Unclear	1 (4.8)	(25)
Performance measures, n (%)		
accuracy	21 (100.0)	(17–19, 23–40)
F1	12 (57.1)	(17, 18, 23–26, 30, 32, 33, 35, 36, 40)
AUC	5 (23.8)	(17, 26, 27, 32, 38)
sensitivity	11 (52.4)	(17, 18, 23, 24, 26, 27, 30, 32, 33, 35, 36)
specificity	8 (38.1)	(18, 26, 27, 30, 32, 33, 35, 36)

Because individual studies may have reported multiple classification tasks, algorithmic models, data types, stress-induction methods, validation approaches, or performance metrics, the totals across categories may exceed the total number of included studies.

### 3.5 Risk of bias and applicability assessment

**Figure 2:**
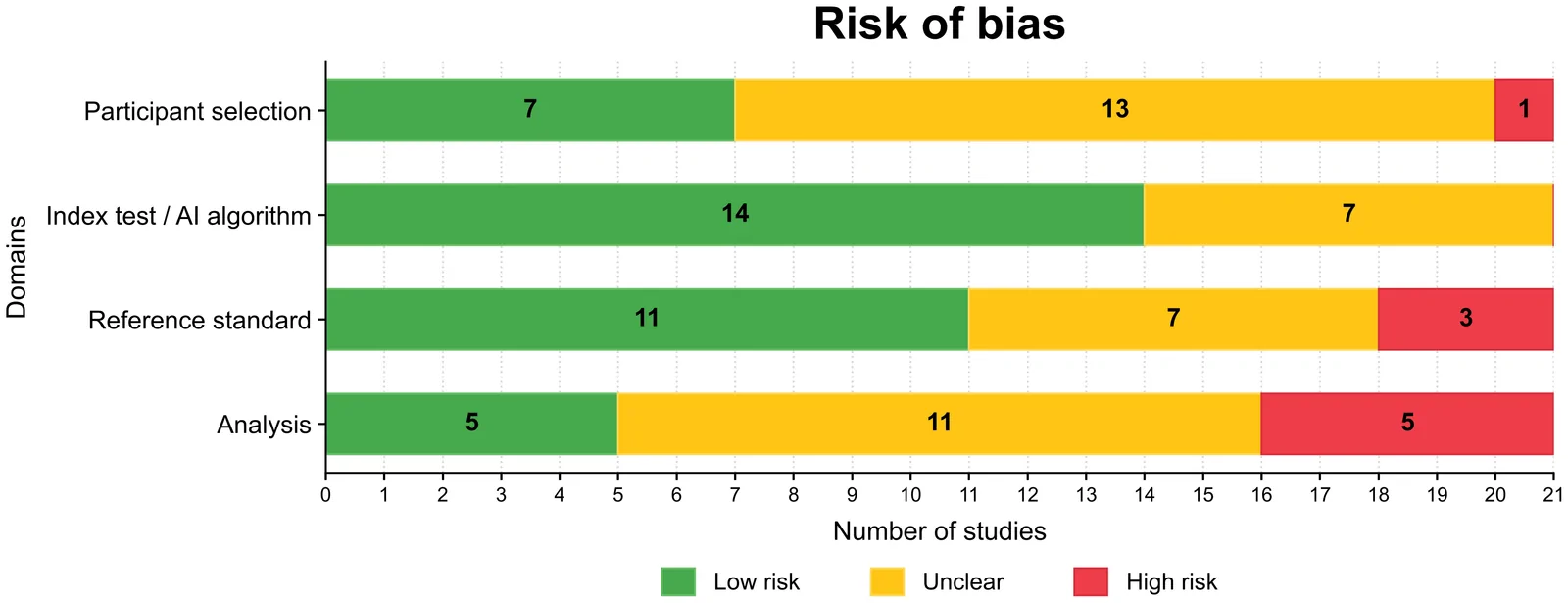
Results of the assessment of risk of bias in the included studies.

**Figure 3:**
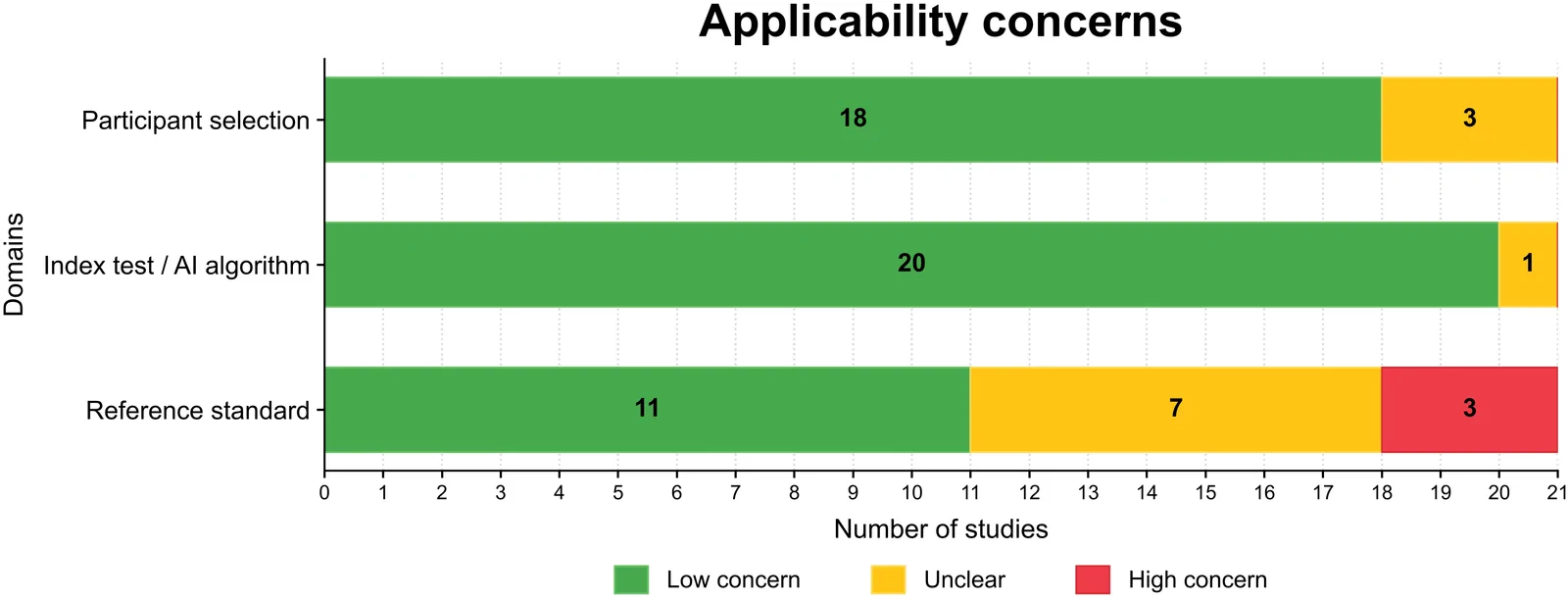
Results of the assessment of applicability concerns in the included studies.

The results of the risk-of-bias and applicability assessments are presented in **Figures 2** and **3**. Overall, relatively high proportions of studies were rated as having a low risk of bias in the index test and reference standard domains, whereas concerns were concentrated primarily in the participant selection and analysis domains.

In the participant selection domain, 7 studies were rated as having a low risk of bias (7/21, 33.3%), 13 as having an unclear risk (13/21, 61.9%), and 1 as having a high risk (1/21, 4.8%). Most studies reported the source of participants and basic eligibility criteria; however, some did not adequately describe their recruitment procedures, sample size justification, or the distribution of participants or observations across stress and nonstress classes. Consequently, most studies were rated as having an unclear risk of bias in this domain. Regarding applicability, 18 studies were rated as having low concern (18/21, 85.7%), indicating that the participant populations in most studies were generally consistent with the review question.

In the index test domain, 14 studies were rated as having a low risk of bias (14/21, 66.7%), and 7 as having an unclear risk (7/21, 33.3%), whereas no studies were rated as having a high risk of bias. Most studies clearly described the procedures for noncontact signal acquisition, preprocessing, feature extraction, and model development. However, some provided insufficient information on feature selection, model training, or the independence of the stress labels from the model inputs. Regarding applicability, 20 studies were rated as having low concern (20/21, 95.2%), suggesting that the model inputs and monitoring approaches used in most studies were consistent with this review’s definition of noncontact physiological or physiology-related signals.

In the reference standard domain, 11 studies were rated as having a low risk of bias (11/21, 52.4%), 7 as having an unclear risk (7/21, 33.3%), and 3 as having a high risk (3/21, 14.3%). Some studies defined stress states according to experimental task phases, self-report questionnaires, or questionnaire scores. Others, however, used cognitive load, clustering results, or indirect physiological indicators as stress labels, creating uncertainty regarding the construct validity of these labels as measures of psychological stress. Regarding applicability, 11 studies were rated as having low concern (11/21, 52.4%), 7 as having unclear concern (7/21, 33.3%), and 3 as having high concern (3/21, 14.3%). These findings indicate that the stress labels used in some studies did not fully correspond to the concept of psychological stress examined in this review.

In the analysis domain, only 5 studies were rated as having a low risk of bias (5/21, 23.8%), whereas 11 were rated as having an unclear risk (11/21, 52.4%) and 5 as having a high risk (5/21, 23.8%). The main concerns were insufficient reporting of whether the training, validation, and test sets were independent at the participant level and whether feature selection, model tuning, and data augmentation were conducted exclusively within the training data. In studies that constructed sample instances from video frames, time windows, or signal segments, the inclusion of different observations from the same participant in both the training and test sets may have resulted in overestimated model performance.

### 3.6 Quantitative findings

#### 3.6.1 Overview

The primary quantitative synthesis was restricted to binary stress classification tasks, with accuracy specified as the primary outcome. Eight studies met the eligibility criteria for the accuracy meta-analysis and were included in the multilevel random-effects model. Of these, 6 studies reported complete confusion matrices, allowing sensitivity, specificity, and F1 score to be calculated and exploratively synthesized as supplementary performance metrics. Studies involving 3-class or multiclass classification were not included in the primary meta-analysis because of their limited number and differences from binary classification studies in class definitions, chance-level classification benchmarks, and task design. Their findings are instead summarized descriptively in the “Other Classification Tasks” section.

#### 3.6.2 Accuracy

**Figure 4:**
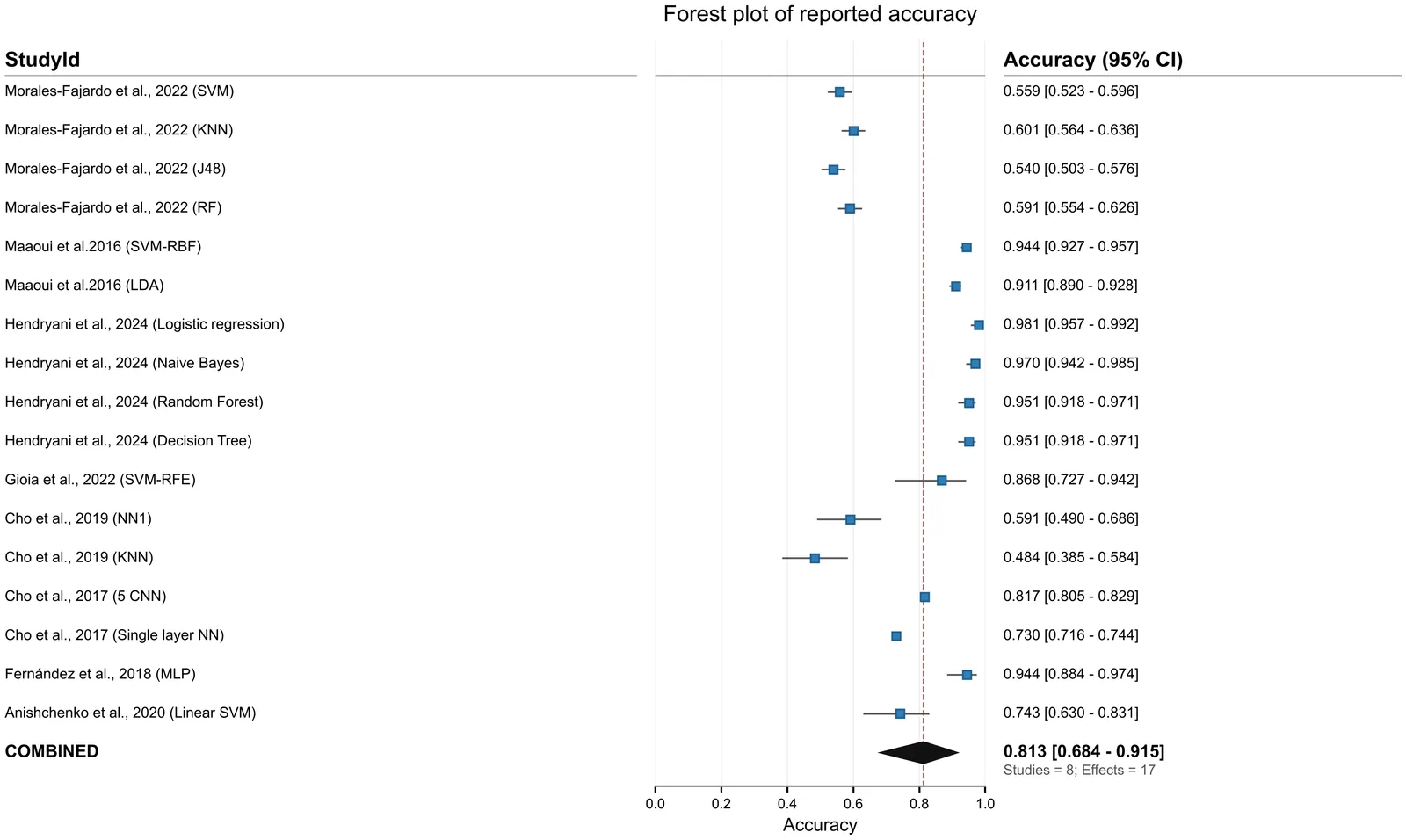
Forest plot of accuracy across included studies. The image presents the accuracy and corresponding 95% confidence intervals for each study (or model). Squares indicate individual effect sizes, with their sizes proportional to study weights, and horizontal lines represent 95% confidence intervals. The diamond indicates the pooled estimate.

A meta-analysis of 17 model-level estimates from 8 studies, comprising 13,910 sample instances, yielded a pooled accuracy of 81.3% (95% CI 68.4%-91.5%; **Figure 4**). Heterogeneity was high, with an approximate total I² of 99.2%. Most of the heterogeneity was attributable to between-study variation (I²=95.6%), whereas within-study or effect estimate–level heterogeneity was relatively small (I²=3.7%).

#### 3.6.3 Other performance metrics

Among the 6 studies reporting complete confusion matrices for binary classification, 11 model-level estimates involving 11,253 sample instances were available for further analysis. Stress was consistently treated as the positive class, and supplementary performance metrics, including F1 score, sensitivity, and specificity, were recalculated from the confusion matrices.

**Figure 5:**
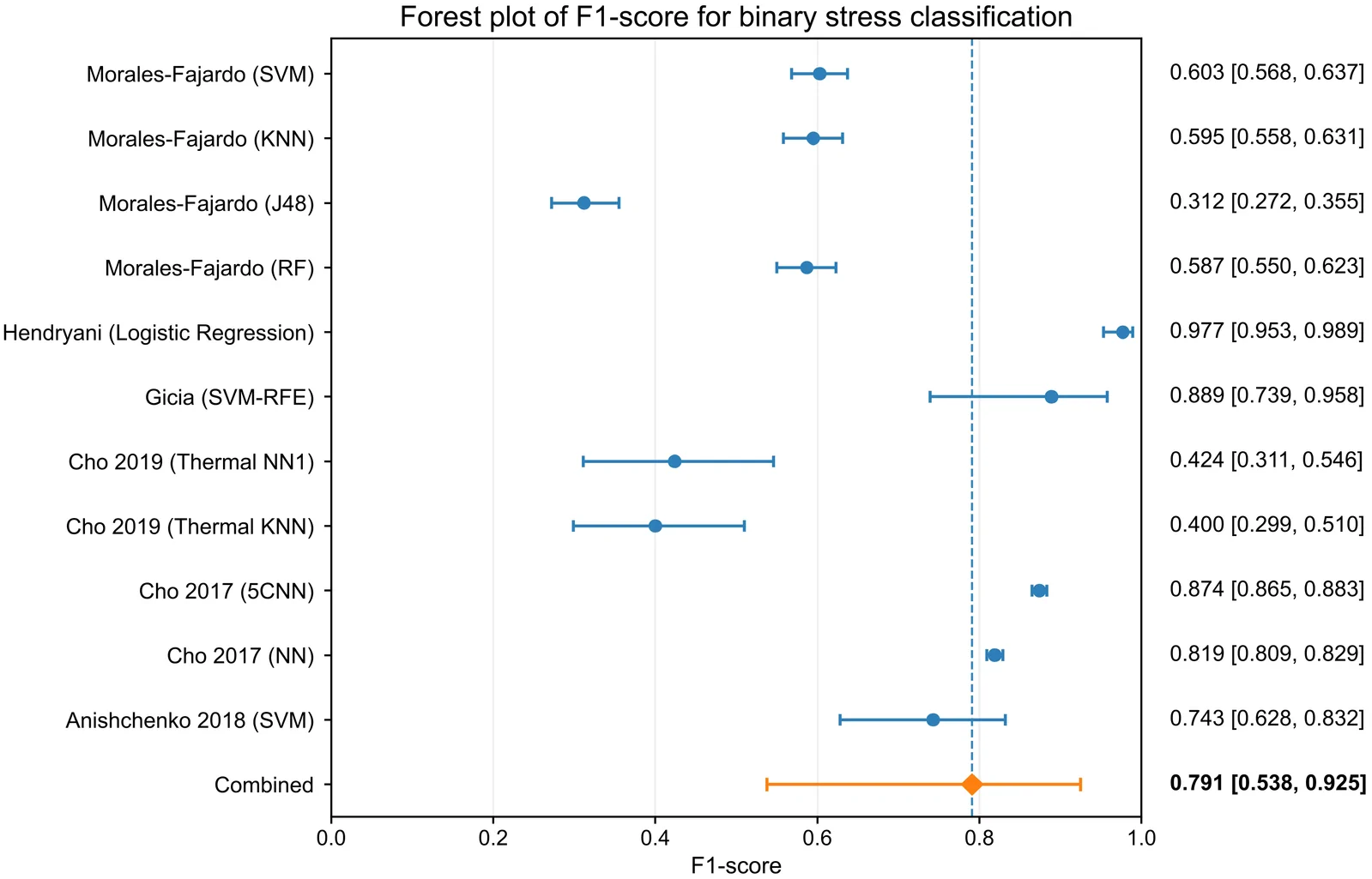
Forest plot of F1 scores for binary stress classification models.

**Figure 6:**
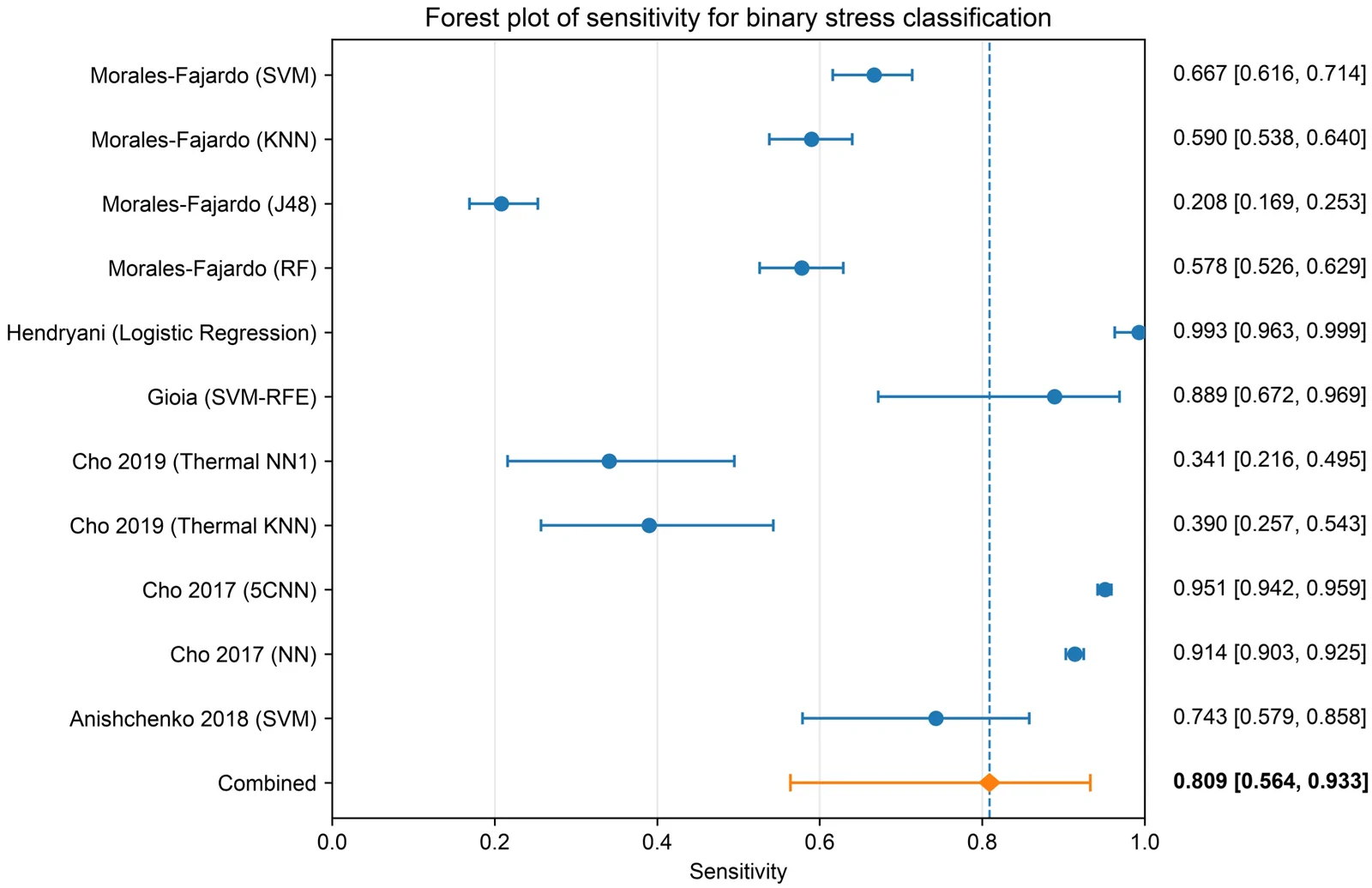
Forest plot of sensitivity for binary stress classification models.

**Figure 7:**
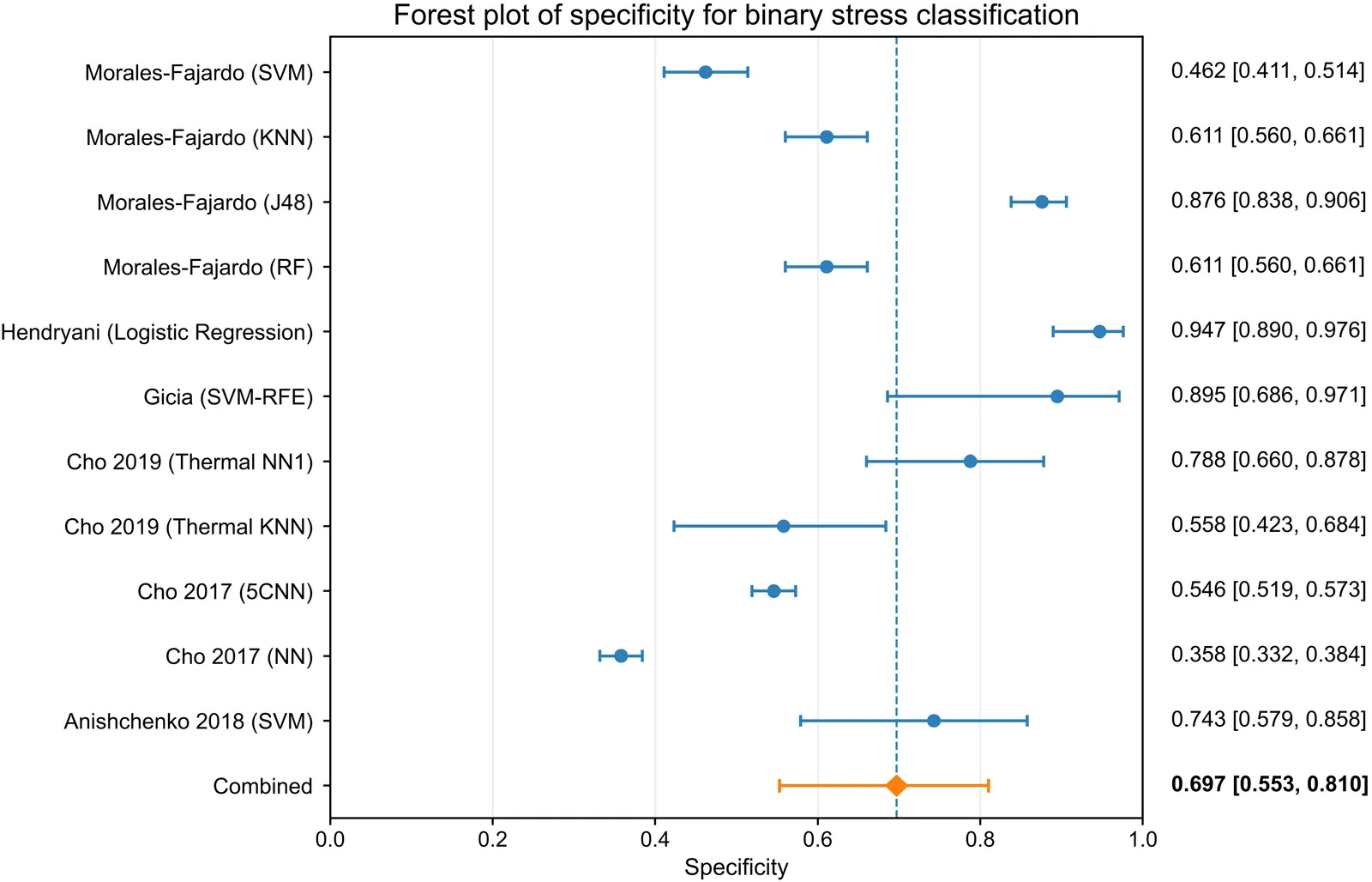
Forest plot of specificity for binary stress classification models.

The pooled F1 score was 0.791 (95% CI 0.538-0.925), with an approximate I² of 99.2%. The pooled sensitivity was 0.809 (95% CI 0.564-0.933), and the pooled specificity was 0.697 (95% CI 0.553-0.810), with an approximate I² of 98.8%. The pooled diagnostic odds ratio was 15.09 (95% CI 1.93-118.16), the positive likelihood ratio was 3.70 (95% CI 1.29-10.57), and the negative likelihood ratio was 0.24 (95% CI 0.08-0.75). The forest plots for F1 score, sensitivity, and specificity are presented in **Figures 5**–**7**.

### 3.7 Subgroup analyses

Exploratory subgroup analyses were conducted according to sensing modality, signal category, source of stress labels, validation approach, and model type. The results are presented in **Table 4**. Differences between subgroups were tested using multilevel meta-regression models.

**Table 4:** Results of the moderator analysis.

Moderator	Studies, n	Effects, n	Sample size	Pooled accuracy, % (95% CI)	*Tau²/Omega²*	*P*
Modality						
rPPG/iPPG	3	10	1847	85.3 (60.6–98.9)	0.052/0.002	0.669
Thermal	3	5	4067	73.1 (44.5–94.0)		
Radar	2	2	178	86.0 (53.5–99.4)		
Signal category						
Heart-rate-related	3	10	1847	85.3 (58.0–99.4)	0.062/0.002	0.680
Facial temperature	2	3	131	70.7 (31.6–97.4)		
Respiration-related	2	3	4044	86.8 (52.6–99.8)		
Label source						
Questionnaire-based	4	9	4773	68.5 (52.3–82.7)	0.021/0.002	0.018
Task-based	4	8	1319	91.2 (79.7–98.3)		
Validation method						
Non-subject-independent or unclear	5	12	2025	85.5 (68.6–96.6)	0.043/0.002	0.334
Subject-independent	3	5	4067	73.0 (47.0–92.6)		
Model type						
SVM	4	4	1690	77.4 (58.9–91.6)	0.052/0.000	0.246
K-nearest neighbor	2	2	799	80.2 (62.3–93.4)		
Decision tree	2	2	971	75.9 (57.1–90.6)		
Random forest	2	2	971	79.0 (60.8–92.7)		
Feed-forward neural networks	3	3	4137	86.6 (68.5–97.8)		

For sensing modality, the pooled accuracies were 85.3% for rPPG/iPPG, 73.1% for thermal imaging, and 86.0% for radar-based sensing. The difference between subgroups was not statistically significant (P = .669). For signal category, the pooled accuracies were 85.3% for heart rate–related signals, 70.7% for facial temperature, and 86.8% for respiration-related signals, with no statistically significant difference between subgroups (P = .680).

Regarding the validation approach, studies using non–participant-independent validation or those with unclear participant-level independence had a pooled accuracy of 85.5%, compared with 73.0% among studies using participant-independent validation. This difference was not statistically significant (P = .334).

For the source of stress labels, the pooled accuracy was 68.5% (95% CI 52.3%-82.7%) for studies using questionnaire-based labels and 91.2% (95% CI 79.7%-98.3%) for studies using task phase–based labels. The between-subgroup difference was statistically significant (P = .018), suggesting that studies defining stress states according to experimental task phases may report higher model performance.

For model type, subgroup analyses were restricted to algorithm categories represented by at least 2 studies. The pooled accuracies were 77.4% for SVMs, 80.2% for k-nearest neighbors, 75.9% for decision trees, 79.0% for random forests, and 86.6% for neural networks. No statistically significant differences were observed across model types (P = .246).

### 3.8 Other classification tasks

Because studies involving 3-class and multiclass classification differed substantially in the number of classes, label definitions, model architectures, and validation approaches, their findings were not statistically pooled. Instead, the ranges of the main model performance metrics were summarized descriptively. Only the primary or final model reported in each study was included in this summary.

All 5 studies reported model accuracy. A total of 5 accuracy estimates were extracted, 1 from the final model of each study. Accuracy ranged from 0.770 to 0.995, with a mean of 0.903 (SD 0.087). Precision, recall, and F1 score were reported in 3 studies. The 3 precision estimates ranged from 0.770 to 0.983, with a mean of 0.873 (SD 0.107). The 3 recall estimates ranged from 0.770 to 0.980, with a mean of 0.872 (SD 0.105), while the 3 F1 score estimates ranged from 0.770 to 0.980, with a mean of 0.871 (SD 0.105).

Other performance metrics were reported inconsistently. Two studies provided receiver operating characteristic area under the curve (ROC-AUC) results. For StressIRNet, class-specific AUC values ranged approximately from 0.89 to 0.98 in the internal test set and from 0.87 to 0.92 in the external validation sets. In the study by Rao et al, the class-specific AUC values for the random forest–based 3-class model ranged approximately from 0.98 to 1.00. Specificity was not clearly reported as a separate summary metric; therefore, its mean and SD were not calculated.

## 4 Discussion

### 4.1 Principal findings

This study systematically evaluated the performance of AI-based psychological stress monitoring using noncontact physiological signals and quantitatively synthesized eligible studies involving binary classification. The findings showed that noncontact AI models achieved moderate-to-high overall performance in binary psychological stress recognition, with a pooled accuracy of 81.3% (95% CI 68.4%-91.5%). In supplementary analyses, the pooled F1 score, sensitivity, and specificity were 0.791, 0.809, and 0.697, respectively, further suggesting that these models have some capacity to discriminate between stress and nonstress states. These findings are broadly consistent with the moderate-to-high classification performance reported in a previous review of AI-based stress monitoring using contact-based or wearable physiological signals (22), indicating that noncontact physiological signals may represent a feasible data source for automated psychological stress monitoring.

Unlike previous approaches that have relied primarily on wearable or contact-based sensors, noncontact sensing places greater emphasis on low user burden, minimal interference, and integration into the surrounding environment. It may therefore have potential applications in workplaces, educational settings, driving environments, remote interactions, and home-based monitoring (41, 42). However, the findings should be interpreted cautiously because relatively few studies were included and substantial heterogeneity was observed across studies. The pooled estimates therefore cannot be directly interpreted as evidence of stable model performance in real-world settings or among previously unseen individuals. Studies involving 3-class and multiclass classification also suggested that noncontact sensing may have the potential to distinguish between different levels of stress. Nevertheless, because of the small number of studies and inconsistencies in task definitions, these findings currently provide only preliminary descriptive evidence.

The subgroup analyses showed no statistically significant differences according to sensing modality, signal category, validation approach, or model type. On the basis of the available evidence, it is therefore not possible to conclude that any particular noncontact sensing method, source of physiological information, validation strategy, or algorithm type provides a consistent performance advantage. Given the small number of studies within each subgroup and the potential for differences in sample size, task design, and label definition across subgroups, these findings should be regarded as exploratory.

Among the subgroup variables examined, only the source of stress labels showed a statistically significant difference. Studies using task phase–based labels reported higher pooled accuracy, consistent with the finding from a previous review of wearable AI for stress monitoring that the definition of the ground truth may influence model accuracy (22). This finding indicates that the operationalization of stress labels is an important factor in interpreting model performance. Task phase–based labeling typically classifies the stress-induction phase as the stress condition and the baseline or resting phase as the nonstress condition. This creates relatively clear class boundaries and may make the classification task less challenging. In contrast, questionnaire- or self-report–based labels categorize stress according to participants’ subjective ratings and may more closely reflect their actual experience of stress. However, these labels are also more susceptible to individual variability, resulting in more complex class boundaries. Consequently, the higher accuracy observed in studies using task phase–based labels may not fully reflect the ability of models to identify the subjective experience of psychological stress; rather, it may partly reflect their ability to distinguish between experimental phases.

In addition to differences in stress-label sources, heterogeneity in stress-induction paradigms should also be considered when interpreting the pooled findings. The included studies used various stress-induction approaches, which are established methods for eliciting psychological stress responses but may differ in stressor characteristics and the psychological processes involved. These differences may lead to variations in physiological response patterns and may partly contribute to the heterogeneity in model performance across studies. Therefore, differences in pooled performance should be interpreted considering not only sensing technologies and algorithmic approaches but also the characteristics of the experimental stress-induction paradigms.

Although the subgroup difference according to validation approach was not statistically significant, studies using non–participant-independent validation or unclear data partitioning reported higher pooled accuracy than those using participant-independent validation. Noncontact signals are often segmented into video frames, time windows, or signal segments that are treated as separate sample instances. If observations from the same participant are included in both the training and test sets, the model may learn participant-specific characteristics rather than stress-related patterns, thereby inflating estimates of model performance.

### 4.2 Research and practical implications

The findings of this review suggest that combining noncontact physiological signals with AI methods has technical potential for automated psychological stress monitoring. However, the current evidence should be regarded primarily as demonstrating technical feasibility rather than providing sufficient support for clinical use or real-world deployment. Psychological stress is multidimensional, dynamic, and highly individualized; therefore, a single model output should not be interpreted as a definitive determination of whether an individual is experiencing stress. A more appropriate application would be to use noncontact stress monitoring as an adjunctive tool for continuous mental health monitoring, risk alerts, or adaptive human-computer interaction. For example, in workplace, educational, driving, and home environments, such systems could track long-term trends to identify unusual increases in an individual’s stress level and provide a basis for subsequent self-assessment, feedback, or intervention.

From a research design perspective, future studies of noncontact stress monitoring should improve their methods and reporting with respect to samples, validation, labeling, and performance evaluation. First, future studies should recruit larger and more representative samples and clearly report participant sources, age, sex, educational level, and the distribution of stress and nonstress classes. This would improve the interpretability and generalizability of findings at the population level.

Second, participant-independent validation should be strengthened. In studies where video frames, time windows, or signal segments are treated as individual sample instances, researchers should clearly report the number of participants, the number of sample instances, class distributions, and the procedures used to divide the data into training, validation, and test sets. Data from the same participant should not be included in both the training and test sets, as this may inflate estimates of model performance.

Third, greater attention should be paid to the construct validity of stress labels. Future studies should avoid directly equating experimental task phases with stress states. Instead, task phases should be reported alongside posttask subjective ratings and, where appropriate, changes in physiological responses. In real-world studies, ecological momentary assessment or repeated-measures designs could be used to better capture dynamic changes in individuals’ perceived stress.

Finally, model performance should be reported more comprehensively. Although accuracy was the most consistently reported metric among the included studies, accuracy alone may not adequately characterize model performance when classes are imbalanced, classification tasks involve multiple stress levels, or the boundary between stress and nonstress is ambiguous. Future studies should therefore report confusion matrices, sensitivity, specificity, precision, recall, F1 score, area under the receiver operating characteristic curve, and class distributions to improve the transparency and comparability of model evaluation.

Overall, the current value of noncontact AI-based stress monitoring lies not in replacing clinical assessments or self-report measures, but in providing a low-burden, continuous, and environmentally embedded form of supportive monitoring. With the incorporation of individualized baseline calibration, multimodal data fusion, longitudinal modeling, and validation in real-world settings, noncontact stress monitoring technologies may play a greater role in digital mental health management.

### 4.3 Limitations

This study has several limitations. First, the number of studies eligible for the primary quantitative synthesis was limited. The primary analysis included only 8 studies involving binary classification, and the supplementary performance analyses were based on even fewer studies. The pooled findings should therefore be regarded as exploratory estimates. Second, substantial methodological heterogeneity existed across the included studies in sensing modalities, stress-induction paradigms, stress-label sources, algorithmic models, and validation strategies. In particular, differences in stress-induction procedures and label definitions may influence how stress states are represented and recognized by AI models. Such heterogeneity may limit direct comparisons between studies and should be considered when interpreting the pooled performance estimates. Third, some primary studies provided insufficient information on data partitioning procedures, participant-level independence, feature selection, and model tuning, which may have resulted in overestimated model performance. Fourth, performance metrics were incompletely reported, particularly confusion matrices, sensitivity, specificity, and AUC, preventing comprehensive synthesis and comparison of all key performance measures.

## 5 Conclusions

This review found that AI models using noncontact physiological signals demonstrated moderate-to-high overall performance in binary psychological stress recognition, while supplementary performance metrics also indicated a degree of discriminatory ability. A small number of studies involving 3-class and multiclass classification further suggested that this technology may have the potential to distinguish between different levels of stress. Compared with contact-based or wearable monitoring, noncontact approaches may offer advantages in terms of low user burden, minimal interference, and integration into the surrounding environment, providing a new technical approach to continuous psychological stress monitoring and digital health management.

However, the current evidence is derived primarily from studies with small samples conducted in laboratory or relatively controlled settings. Considerable variation also exists in stress labels, sensing modalities, validation strategies, and the reporting of performance metrics. At present, noncontact AI-based stress monitoring should therefore be regarded as evidence of technical feasibility and as a potential adjunctive monitoring tool rather than a mature method for individual-level diagnosis. Future research should prioritize the standardization of stress labels, participant-level independent validation, comprehensive reporting of performance metrics, and validation in real-world settings to advance the development of more interpretable, reproducible, and practically applicable digital mental health tools.

## Data Availability

The original contributions presented in the study are included in the article/**Supplementary Material**. Further inquiries can be directed to the corresponding author.
