# Supplementary material for: Performance of artificial intelligence models for monitoring psychological stress using noncontact physiological signals: a systematic review and meta-analysis
Source: Front Psychiatry. 2026 Aug 19;17:1938767. doi: 10.3389/fpsyt.2026.1938767 (PMC13534124; doi:10.3389/fpsyt.2026.1938767)
Supplement: Supplementary file 1 [file SupplementaryFile1.docx]

**Modified QUADAS-2 Criteria for Risk-of-Bias and Applicability Assessment**

This study used a modified Quality Assessment of Diagnostic Accuracy Studies–2 (QUADAS-2) tool to evaluate the risk of bias and applicability of the included studies. The assessment tool included four risk-of-bias domains: participants, index test, reference standard, and analysis. Applicability concerns were assessed in three domains: participants, index test, and reference standard. Each domain included several signaling questions, which were rated as “yes,” “no,” or “unclear” according to the information reported in each study. After considering all signaling questions within each domain, the risk of bias was judged as low, some concerns/unclear, or high. Applicability was judged as low concern, some concerns/unclear, or high concern.

1. Participants: Participant Selection

1.1 Were participants recruited consecutively or randomly?

Yes: The study clearly reported consecutive recruitment, random sampling, or systematic recruitment from the eligible target population.

No: The study clearly used a convenience sample, such as laboratory students, volunteers, students from a specific course, or participants accessible to the research team, without explaining the representativeness of the sample.

Unclear: The recruitment method was not reported.

1.2 Were inappropriate exclusions avoided?

Yes: The inclusion and exclusion criteria were reasonable, with no obvious exclusion of specific subgroups that would affect model applicability. If participants were excluded because of device failure, poor video quality, or inability to extract signals, the study reported the reasons for exclusion and the number of excluded participants.

No: Inappropriate exclusions were present, such as including only participants with clear stress responses, including only participants with good signal quality, excluding participants with more motion artifacts without adequate explanation, or excluding specific populations that could substantially affect model performance.

Unclear: The inclusion and exclusion criteria were not reported, or the reasons for data exclusion were not described.

1.3 Was the sample size sufficient?

Yes: For model validation studies, the number of participants with outcomes was at least 100; alternatively, if multiple samples were recorded from each participant and machine learning (ML) algorithms used sample-level rather than participant-level inputs, the total number of samples was at least 100.

No: For model validation studies, the number of participants with outcomes was less than 100, and, in cases where multiple samples were recorded from each participant and ML algorithms used sample-level inputs, the total number of samples was also less than 100.

Unclear: For model development studies, information on the number of candidate predictors or the number of participants with outcomes was insufficient to calculate the number of events per variable (EPV). For model validation studies, information on the number of participants with outcomes was insufficient.

1.4 Were the stress and nonstress groups generally balanced?

Yes: The stress and nonstress conditions were generally balanced at the participant or state level. In paired designs, if each participant completed both stress and nonstress conditions, the groups were considered generally balanced.

No: One class accounted for more than two-thirds of the total sample, or the classes were severely imbalanced without appropriate handling.

Unclear: The study did not report the number of participants, states, or samples in each class.

Judgment of Risk of Bias in the Participants Domain

Low risk: All or most signaling questions were rated as “yes,” and the participant source, inclusion and exclusion criteria, number of independent participants, and class distribution were clearly reported.

Some concerns/unclear: The study used a convenience sample, had a small sample size, did not clearly report the recruitment method, did not fully report class distributions, or excluded some data because of signal quality issues with reasons that were considered acceptable.

High risk: The number of independent participants was extremely small; there was clear selective inclusion or inappropriate exclusion; only participants with clear stress responses were included; or a large number of windows, segments, or feature vectors were used to mask an insufficient number of independent participants.

Judgment of Applicability in the Participants Domain

Low concern: Participants were healthy adults, university students, general workers, or other target populations generally relevant to psychological stress recognition.

Some concerns/unclear: The sample came from a narrow or specific setting, such as students from a single major, employees from a single company, participants in a driving simulator, or a very small sample of healthy volunteers, but was still relevant to psychological stress recognition.

High concern: The study population was clearly not representative of the target population for psychological stress recognition, or the study mainly focused on disease status, deception detection, gaming disorder, competitive sports, or other special states that were difficult to generalize to the review question.

2. Index Test: Noncontact Signals and AI Models

2.1 Was the AI model clearly described?

Yes: The study clearly reported the model type, inputs, outputs, main parameters, training procedure, and classification task. For deep learning models, the study reported basic information such as the network architecture, number of layers, activation functions, optimizer, and number of training epochs. For traditional ML models, the study reported the classifier type, kernel function, or main parameters.

No: The study only reported the model name, such as “SVM” or “CNN,” without sufficient information on key parameters, inputs and outputs, or the training procedure.

Unclear: The model description was insufficient to determine the training and prediction process.

2.2 Were the input signals and features clearly described?

Yes: The study clearly reported all input signals and features, such as remote or imaging photoplethysmography (rPPG/iPPG), thermal imaging, radar, ballistocardiography (BCG), WiFi channel state information (CSI), heart rate (HR), heart rate variability (HRV), respiration, temperature regions of interest (ROIs), and facial landmarks, and specified which features were used in each model.

No: The input features were not clearly described; or although the overall feature set was reported, it was not possible to determine which features were used in each model.

Unclear: The signal source or feature definitions were insufficiently reported.

2.3 Were feature acquisition and extraction consistent across all participants?

Yes: All participants were assessed using the same device, acquisition procedure, ROI definition, preprocessing method, and feature extraction method.

No: Different devices, acquisition conditions, or feature definitions were used across participants, or subjective ROI or feature extraction was performed by different assessors without controlling for consistency.

Unclear: The study did not adequately describe the feature acquisition or extraction procedures.

2.4 Was feature extraction independent of the stress labels?

Yes: Signal preprocessing, ROI selection, and feature extraction were prespecified or automated and did not use stress or nonstress label information. If feature selection was performed, it was nested within the training set or within the training folds of cross-validation.

No: Feature selection, ROI selection, threshold setting, or model input selection clearly used label information from the full dataset; or the study first compared stress and nonstress differences in the full sample and then used statistically significant features for cross-validated classification.

Unclear: The study did not report whether feature selection or ROI selection was performed within the training set.

Judgment of Risk of Bias in the Index Test Domain

Low risk: The noncontact signals, input features, AI model, and feature extraction procedure were clearly described, and there was no evidence of label-dependent feature selection.

Some concerns/unclear: The model and features were generally clear, but some parameters, feature selection procedures, or ROI processing methods were insufficiently reported; or the best-performing model combined noncontact signals with other behavioral features, but results for models based on noncontact signals could be extracted separately.

High risk: The model or features were poorly reported; feature selection was clearly based on labels from the full dataset; or the final model mainly relied on nontarget signals, and results based on noncontact physiological signals could not be separated.

Judgment of Applicability in the Index Test Domain

Low concern: The model inputs were mainly noncontact physiological or physiology-related signals, such as rPPG/iPPG, thermal imaging, radar, millimeter-wave signals, or noncontact respiration or heart rate signals.

Some concerns/unclear: The model combined noncontact signals with contact-based signals, demographic variables, facial expressions, facial landmarks, or behavioral features, but results for the noncontact model could be extracted separately.

High concern: The model mainly relied on contact-based signals, questionnaire variables, demographic variables, or behavioral features; noncontact signals were only auxiliary; or results for noncontact signals could not be separated.

3. Reference Standard: Stress Labels

3.1 Was the reference standard likely to correctly distinguish stress states?

Yes: Stress labels were derived from standard or clearly defined stress-induction tasks, such as the Trier Social Stress Test (TSST), Stroop task, mental arithmetic task, social evaluation task, or examination stress task; or from validated psychological scales, clinical assessment, psychologist assessment, or clearly defined task phases, and the label definition was relevant to psychological stress.

No: Stress labels were generated using physiological rules that substantially overlapped with the model inputs, such as HR, HRV, respiration, skin temperature, or thermal imaging ROI intensity; or the labels actually represented disease status, tachycardia, gaming duration, deception detection, or other outcomes not corresponding to psychological stress.

Unclear/some concerns: Labels were derived from a single-item visual analog scale (VAS), single subjective rating, individualized normalized clustering, k-means clustering, NASA Task Load Index (NASA-TLX), State-Trait Anxiety Inventory state anxiety scores, or other proxy indicators of stress. These labels were related to stress but were not strict standard stress labels.

3.2 Was the label definition consistent across all participants?

Yes: All participants were labeled using the same task, scale, threshold, or annotation rule.

No: Different label definitions, thresholds, or tasks were used across participants, or the labeling rule varied by participant without reasonable justification.

Unclear: The label definition, threshold, or annotation procedure was not reported.

3.3 Was label determination independent of the model input features?

Yes: Stress labels were determined by task phase, scale score, self-report, expert assessment, or an external standard and did not depend on model input signals.

No: Labels were generated using physiological signals that were the same as or highly overlapping with the model inputs; for example, thermal imaging ROI temperature changes were used to define stress levels, and a thermal imaging model was then trained to recognize those levels; or HR, HRV, or respiration-based rules were used to generate labels, and rPPG, radar, or thermal physiological signals were then used for prediction.

Unclear: The study did not report whether label generation used model input features.

3.4 Did the label time window match the index test?

Yes: Stress labels corresponded to the time window of noncontact signal acquisition; for example, signals collected during a task or immediately after a task were matched with concurrent stress labels, or the time window of scale assessment was generally consistent with the signal acquisition period.

No: The label assessment window was clearly inconsistent with the model input signals; for example, a stress scale referring to the past month was used to label physiological signals collected over only a few minutes, without adequate justification.

Unclear: The temporal correspondence between labels and signal acquisition was not reported.

Judgment of Risk of Bias in the Reference Standard Domain

Low risk: Stress labels were derived from standard tasks, clearly defined task phases, or validated scales; label definitions were consistent; labels were independent of model inputs; and the time windows were matched.

Some concerns/unclear: Labels were derived from single-item self-reports, VAS scores, clustering-based labels, workload scales, state anxiety scales, or behavioral proxy labels; or label definitions and time windows were insufficiently reported.

High risk: Labels were generated from physiological indicators, thermal imaging intensity rules, or model features that highly overlapped with the model inputs; or the study outcome was not psychological stress.

Judgment of Applicability in the Reference Standard Domain

Low concern: Labels clearly corresponded to psychological stress or acute stress states, such as stress versus nonstress, rest versus stress task, before and after TSST, or before and after Stroop or mental arithmetic stress tasks.

Some concerns/unclear: Labels represented state anxiety, VAS-based clusters, subjective stress levels, or daily stress levels. Although these labels were related to psychological stress, they did not fully correspond to a standard stress versus nonstress definition.

High concern: Labels represented nonstress outcomes, such as deception detection, disease status, gaming disorder, behavioral duration, heart rate abnormalities, or “stress levels” generated using physiological rules.

4. Analysis: Data Analysis Process

4.1 Were all included participants entered into the analysis?

Yes: All included participants were entered into the final analysis, or exclusions were clearly described, reasonable, and limited in number.

No: A large number of participants were excluded because of signal quality, motion artifacts, missing labels, or other reasons, and the study did not explain whether these exclusions could introduce bias; or only samples with better classification performance were retained.

Unclear: The final number of participants included in the analysis or the reasons for exclusion were not reported.

4.2 Were preprocessing and missing data handling appropriate?

Yes: The study clearly reported signal preprocessing, artifact handling, outlier handling, and missing data handling methods, and these methods were considered reasonable. Data augmentation, normalization, or filtering was appropriately implemented within the training process.

No: Missing data were directly deleted without explanation; outlier removal substantially affected the results; data augmentation or normalization may have leaked information across the training and test sets; or the preprocessing procedure was clearly inappropriate.

Unclear: Preprocessing, missing data handling, or outlier handling methods were insufficiently reported.

4.3 Were the training, validation, and test sets appropriately split and participant-independent?

Yes: The study clearly used participant-independent validation, such as leave-one-subject-out validation, subject-wise k-fold cross-validation, subject-wise hold-out validation, or external validation. All windows, segments, ROIs, images, and state samples from the same participant appeared only in either the training set or the test set, but not both.

No: The study clearly used random splitting at the window, segment, image, ROI, or sample level, allowing data from the same participant to appear in both the training and test sets; or feature selection or model selection was performed on the full dataset before cross-validation.

Unclear: The study reported only k-fold cross-validation, hold-out split, or train/test split, without specifying whether the split was performed at the participant level.

4.4 Was model performance adequately evaluated?

Yes: The study reported a confusion matrix or multiple appropriate metrics, such as accuracy, sensitivity/recall, specificity, precision, F1 score, and AUC; and the metrics were appropriate for the classification task.

No: Only accuracy was reported; no confusion matrix or other classification metrics were reported; or the metric definitions were unclear, making it difficult to evaluate model performance.

Unclear: Performance metrics were insufficiently reported, or the calculation method could not be confirmed.

Judgment of Risk of Bias in the Analysis Domain

Low risk: The analyzed sample was clearly reported; preprocessing was appropriate; the training, validation, and test sets were clearly participant-independent; and model performance metrics were adequately reported.

Some concerns/unclear: Ordinary k-fold cross-validation or train/test split was used without specifying whether the split was subject-wise; preprocessing or feature selection procedures were not fully clear; or only a limited set of performance metrics was reported, but the main results could still be extracted.

High risk: Random splitting was clearly performed at the sample, window, segment, ROI, or image level; data from the same participant may have appeared in both the training and test sets; feature selection was not nested within cross-validation; or only training-set performance or severely insufficient performance metrics were reported.
