# Supplementary material for: Performance of artificial intelligence models for monitoring psychological stress using noncontact physiological signals: a systematic review and meta-analysis
Source: Front Psychiatry. 2026 Aug 19;17:1938767. doi: 10.3389/fpsyt.2026.1938767 (PMC13534124; doi:10.3389/fpsyt.2026.1938767)
Supplement: Supplementary file 2 [file SupplementaryFile2.docx]

Monday, May 30, 2022 6:45:26 AM Monday, May 30, 2022 6:39:40 AM

**Multimedia Appendix 2: Search Strategy**

| **Database** | **Query** | **Results** |
| --- | --- | --- |
| **PubMed** | (  "Stress, Psychological"[Mesh]  OR stress[tiab]  OR "mental stress"[tiab]  OR "psychological stress"[tiab]  OR "perceived stress"[tiab]  OR "emotional stress"[tiab]  OR "subjective stress"[tiab]  OR "acute stress"[tiab]  OR "cognitive stress"[tiab]  OR "psychosocial stress"[tiab]  OR "stress level"[tiab]  OR "stress levels"[tiab]  OR "stress state"[tiab]  OR "stress states"[tiab]  )  AND  (  detect*[tiab]  OR recogni*[tiab]  OR classif*[tiab]  OR predict*[tiab]  OR monitor*[tiab]  OR identif*[tiab]  OR assess*[tiab]  OR estimat*[tiab]  OR discriminat*[tiab]  )  AND  (  noncontact[tiab]  OR "non-contact"[tiab]  OR contactless[tiab]  OR unobtrusive[tiab]  OR unobtrus*[tiab]  OR "camera-based"[tiab]  OR "video-based"[tiab]  OR webcam[tiab]  OR "facial video"[tiab]  OR "infrared camera"[tiab]  OR "infrared cameras"[tiab]  OR "thermal camera"[tiab]  OR "thermal cameras"[tiab]  OR "thermal imaging"[tiab]  OR thermography[tiab]  OR radar[tiab]  OR "Doppler radar"[tiab]  OR FMCW[tiab]  OR "CW radar"[tiab]  OR "millimeter wave"[tiab]  OR mmWave[tiab]  OR microwave[tiab]  OR UWB[tiab]  OR "ultra-wideband"[tiab]  OR WiFi[tiab]  OR "WiFi CSI"[tiab]  OR "wireless sensing"[tiab]  OR RFID[tiab]  OR rPPG[tiab]  OR iPPG[tiab]  OR "remote photoplethysmography"[tiab]  OR "remote photoplethysmographic"[tiab]  OR "imaging photoplethysmography"[tiab]  OR "imaging photoplethysmographic"[tiab]  OR "bed sensor"[tiab]  OR "bed sensors"[tiab]  OR "smart bed"[tiab]  OR "smart beds"[tiab]  OR mattress[tiab]  OR "bed-based"[tiab]  OR piezoelectric[tiab]  OR "pressure sensor"[tiab]  OR "pressure sensors"[tiab]  OR "smart chair"[tiab]  OR "smart chairs"[tiab]  OR "seat sensor"[tiab]  OR "seat sensors"[tiab]  OR "chair sensor"[tiab]  OR "chair sensors"[tiab]  OR "seat cushion"[tiab]  OR "seat cushions"[tiab]  OR "load cell"[tiab]  OR "load cells"[tiab]  )  AND  (  physiolog*[tiab]  OR biosignal*[tiab]  OR biopotential*[tiab]  OR "vital sign"[tiab]  OR "vital signs"[tiab]  OR autonomic[tiab]  OR cardiorespiratory[tiab]  OR PPG[tiab]  OR photoplethysmogra*[tiab]  OR rPPG[tiab]  OR iPPG[tiab]  OR "remote photoplethysmography"[tiab]  OR "remote photoplethysmographic"[tiab]  OR "imaging photoplethysmography"[tiab]  OR "imaging photoplethysmographic"[tiab]  OR BVP[tiab]  OR "blood volume pulse"[tiab]  OR BCG[tiab]  OR ballistocardiogra*[tiab]  OR "heart rate"[tiab]  OR HRV[tiab]  OR "heart rate variability"[tiab]  OR PRV[tiab]  OR "pulse rate variability"[tiab]  OR IBI[tiab]  OR "interbeat interval"[tiab]  OR "interbeat intervals"[tiab]  OR respiration[tiab]  OR respiratory[tiab]  OR "respiratory rate"[tiab]  OR "skin temperature"[tiab]  OR "body temperature"[tiab]  OR SpO2[tiab]  OR "oxygen saturation"[tiab]  OR PWA[tiab]  OR "pulse wave amplitude"[tiab]  )  AND  (  "Artificial Intelligence"[Mesh]  OR "Machine Learning"[Mesh]  OR "Deep Learning"[Mesh]  OR "artificial intelligence"[tiab]  OR "machine learning"[tiab]  OR "deep learning"[tiab]  OR "supervised learning"[tiab]  OR "ensemble learning"[tiab]  OR "pattern recognition"[tiab]  OR classifier*[tiab]  OR "support vector machine"[tiab]  OR "support vector machines"[tiab]  OR SVM[tiab]  OR "random forest"[tiab]  OR "random forests"[tiab]  OR "decision tree"[tiab]  OR "decision trees"[tiab]  OR "k-nearest neighbor"[tiab]  OR "k-nearest neighbors"[tiab]  OR KNN[tiab]  OR "artificial neural network"[tiab]  OR "artificial neural networks"[tiab]  OR "convolutional neural network"[tiab]  OR "convolutional neural networks"[tiab]  OR "recurrent neural network"[tiab]  OR "recurrent neural networks"[tiab]  OR "long short-term memory"[tiab]  OR LSTM[tiab]  OR CNN[tiab]  OR XGBoost[tiab]  OR AdaBoost[tiab]  OR "logistic regression"[tiab]  OR "naive bayes"[tiab]  OR "linear discriminant analysis"[tiab]  OR LDA[tiab]  )  NOT  (  "oxidative stress"[tiab]  OR "heat stress"[tiab]  OR "thermal stress"[tiab]  OR "mechanical stress"[tiab]  OR "cell stress"[tiab]  OR "plant stress"[tiab]  OR "crop stress"[tiab]  OR "leaf stress"[tiab]  OR "drought stress"[tiab]  OR "salt stress"[tiab]  OR "water stress"[tiab]  OR "stress-strain"[tiab]  )  NOT  (  review[pt]  OR systematic review[pt]  OR meta-analysis[pt]  OR protocol[ti]  ) | 50 |
| **IEEE Xplore** | (  (stress OR "mental stress" OR "psychological stress" OR "perceived stress" OR "emotional stress" OR "subjective stress" OR "stress level" OR "stress levels" OR "stress state" OR "stress states") <in> Metadata  )  AND  (  (detect OR detects OR detected OR detecting OR detection OR recognition OR recognize OR recognizing OR recognised OR classify OR classifies OR classified OR classification OR monitor OR monitoring OR identify OR identification OR assess OR assessment OR estimate OR estimation OR discriminate OR discrimination OR predict OR prediction) <in> Metadata  )  AND  (  (noncontact OR "non-contact" OR contactless OR unobtrusive OR "camera-based" OR "video-based" OR webcam OR "facial video" OR "infrared camera" OR "infrared cameras" OR "thermal camera" OR "thermal cameras" OR "thermal imaging" OR thermography OR radar OR "Doppler radar" OR FMCW OR "CW radar" OR "millimeter wave" OR mmWave OR microwave OR UWB OR "ultra-wideband" OR WiFi OR "WiFi CSI" OR "wireless sensing" OR RFID OR rPPG OR iPPG OR "remote photoplethysmography" OR "remote photoplethysmographic" OR "imaging photoplethysmography" OR "imaging photoplethysmographic" OR "bed sensor" OR "bed sensors" OR "smart bed" OR "smart beds" OR mattress OR "bed-based" OR piezoelectric OR "pressure sensor" OR "pressure sensors" OR "smart chair" OR "smart chairs" OR "seat sensor" OR "seat sensors" OR "chair sensor" OR "chair sensors" OR "seat cushion" OR "seat cushions" OR "load cell" OR "load cells") <in> Metadata  )  AND  (  (physiological OR physiology OR biosignal OR biosignals OR biopotential OR biopotentials OR "vital sign" OR "vital signs" OR autonomic OR cardiorespiratory OR PPG OR photoplethysmography OR photoplethysmographic OR rPPG OR iPPG OR "remote photoplethysmography" OR "remote photoplethysmographic" OR "imaging photoplethysmography" OR "imaging photoplethysmographic" OR BVP OR "blood volume pulse" OR BCG OR ballistocardiography OR ballistocardiographic OR "heart rate" OR HRV OR "heart rate variability" OR PRV OR "pulse rate variability" OR IBI OR "interbeat interval" OR "interbeat intervals" OR respiration OR respiratory OR "respiratory rate" OR "skin temperature" OR "body temperature" OR SpO2 OR "oxygen saturation" OR PWA OR "pulse wave amplitude") <in> Metadata  )  AND  (  ("artificial intelligence" OR "machine learning" OR "deep learning" OR "supervised learning" OR "ensemble learning" OR "support vector machine" OR "support vector machines" OR SVM OR "random forest" OR "random forests" OR "decision tree" OR "decision trees" OR "k-nearest neighbor" OR "k-nearest neighbors" OR KNN OR "artificial neural network" OR "artificial neural networks" OR "neural network" OR "neural networks" OR "convolutional neural network" OR "convolutional neural networks" OR "recurrent neural network" OR "recurrent neural networks" OR "long short-term memory" OR LSTM OR CNN OR XGBoost OR AdaBoost OR "pattern recognition" OR classifier OR classifiers OR "linear discriminant analysis" OR LDA OR "logistic regression" OR "naive bayes") <in> Metadata  )  NOT  (  ("oxidative stress" OR "heat stress" OR "thermal stress" OR "mechanical stress" OR "cell stress" OR "plant stress" OR "crop stress" OR "leaf stress" OR "drought stress" OR "salt stress" OR "water stress" OR "systematic review" OR "meta-analysis" OR "review article" OR "survey paper" OR protocol) <in> Metadata  ) | 1 |
| **EMBASE** | (  'psychological stress'/exp  OR stress:ti,ab,kw  OR 'mental stress':ti,ab,kw  OR 'psychological stress':ti,ab,kw  OR 'perceived stress':ti,ab,kw  OR 'emotional stress':ti,ab,kw  OR 'subjective stress':ti,ab,kw  OR 'acute stress':ti,ab,kw  OR 'cognitive stress':ti,ab,kw  OR 'psychosocial stress':ti,ab,kw  OR 'stress level*':ti,ab,kw  OR 'stress state*':ti,ab,kw  )  AND  (  detect*:ti,ab,kw  OR recogni*:ti,ab,kw  OR classif*:ti,ab,kw  OR predict*:ti,ab,kw  OR monitor*:ti,ab,kw  OR identif*:ti,ab,kw  OR assess*:ti,ab,kw  OR estimat*:ti,ab,kw  OR discriminat*:ti,ab,kw  )  AND  (  noncontact:ti,ab,kw  OR 'non-contact':ti,ab,kw  OR contactless:ti,ab,kw  OR unobtrusive:ti,ab,kw  OR unobtrus*:ti,ab,kw  OR 'camera-based':ti,ab,kw  OR 'video-based':ti,ab,kw  OR webcam:ti,ab,kw  OR 'facial video':ti,ab,kw  OR 'infrared camera*':ti,ab,kw  OR 'thermal camera*':ti,ab,kw  OR 'thermal imaging':ti,ab,kw  OR thermography:ti,ab,kw  OR radar:ti,ab,kw  OR 'doppler radar':ti,ab,kw  OR FMCW:ti,ab,kw  OR 'CW radar':ti,ab,kw  OR 'millimeter wave':ti,ab,kw  OR mmWave:ti,ab,kw  OR microwave:ti,ab,kw  OR UWB:ti,ab,kw  OR 'ultra-wideband':ti,ab,kw  OR WiFi:ti,ab,kw  OR 'WiFi CSI':ti,ab,kw  OR 'wireless sensing':ti,ab,kw  OR RFID:ti,ab,kw  OR rPPG:ti,ab,kw  OR iPPG:ti,ab,kw  OR 'remote photoplethysmograph*':ti,ab,kw  OR 'remote photoplethysmography':ti,ab,kw  OR 'remote photoplethysmographic':ti,ab,kw  OR 'imaging photoplethysmograph*':ti,ab,kw  OR 'imaging photoplethysmography':ti,ab,kw  OR 'imaging photoplethysmographic':ti,ab,kw  OR 'bed sensor*':ti,ab,kw  OR 'smart bed*':ti,ab,kw  OR mattress:ti,ab,kw  OR 'bed-based':ti,ab,kw  OR piezoelectric:ti,ab,kw  OR 'pressure sensor*':ti,ab,kw  OR 'smart chair*':ti,ab,kw  OR 'seat sensor*':ti,ab,kw  OR 'chair sensor*':ti,ab,kw  OR 'seat cushion*':ti,ab,kw  OR 'load cell*':ti,ab,kw  )  AND  (  physiolog*:ti,ab,kw  OR biosignal*:ti,ab,kw  OR biopotential*:ti,ab,kw  OR 'vital sign*':ti,ab,kw  OR autonomic:ti,ab,kw  OR cardiorespiratory:ti,ab,kw  OR PPG:ti,ab,kw  OR photoplethysmogra*:ti,ab,kw  OR rPPG:ti,ab,kw  OR iPPG:ti,ab,kw  OR 'remote photoplethysmograph*':ti,ab,kw  OR 'remote photoplethysmography':ti,ab,kw  OR 'remote photoplethysmographic':ti,ab,kw  OR 'imaging photoplethysmograph*':ti,ab,kw  OR 'imaging photoplethysmography':ti,ab,kw  OR 'imaging photoplethysmographic':ti,ab,kw  OR BVP:ti,ab,kw  OR 'blood volume pulse':ti,ab,kw  OR BCG:ti,ab,kw  OR ballistocardiogra*:ti,ab,kw  OR 'heart rate':ti,ab,kw  OR HRV:ti,ab,kw  OR 'heart rate variability':ti,ab,kw  OR PRV:ti,ab,kw  OR 'pulse rate variability':ti,ab,kw  OR IBI:ti,ab,kw  OR 'interbeat interval*':ti,ab,kw  OR respiration:ti,ab,kw  OR respiratory:ti,ab,kw  OR 'respiratory rate':ti,ab,kw  OR 'skin temperature':ti,ab,kw  OR 'body temperature':ti,ab,kw  OR SpO2:ti,ab,kw  OR 'oxygen saturation':ti,ab,kw  OR PWA:ti,ab,kw  OR 'pulse wave amplitude':ti,ab,kw  )  AND  (  'artificial intelligence'/exp  OR 'machine learning'/exp  OR 'deep learning'/exp  OR 'artificial intelligence':ti,ab,kw  OR 'machine learning':ti,ab,kw  OR 'deep learning':ti,ab,kw  OR 'supervised learning':ti,ab,kw  OR 'ensemble learning':ti,ab,kw  OR 'pattern recognition':ti,ab,kw  OR classifier*:ti,ab,kw  OR 'classification model*':ti,ab,kw  OR 'predictive model*':ti,ab,kw  OR 'support vector machine*':ti,ab,kw  OR SVM:ti,ab,kw  OR 'random forest*':ti,ab,kw  OR 'decision tree*':ti,ab,kw  OR 'k-nearest neighbor*':ti,ab,kw  OR KNN:ti,ab,kw  OR 'artificial neural network*':ti,ab,kw  OR 'neural network*':ti,ab,kw  OR 'convolutional neural network*':ti,ab,kw  OR 'recurrent neural network*':ti,ab,kw  OR 'long short-term memory':ti,ab,kw  OR LSTM:ti,ab,kw  OR CNN:ti,ab,kw  OR XGBoost:ti,ab,kw  OR AdaBoost:ti,ab,kw  OR 'logistic regression':ti,ab,kw  OR 'naive bayes':ti,ab,kw  OR 'linear discriminant analysis':ti,ab,kw  OR LDA:ti,ab,kw  )  NOT  (  'oxidative stress':ti,ab,kw  OR 'heat stress':ti,ab,kw  OR 'thermal stress':ti,ab,kw  OR 'mechanical stress':ti,ab,kw  OR 'cell stress':ti,ab,kw  OR 'plant stress':ti,ab,kw  OR 'crop stress':ti,ab,kw  OR 'leaf stress':ti,ab,kw  OR 'drought stress':ti,ab,kw  OR 'salt stress':ti,ab,kw  OR 'water stress':ti,ab,kw  OR 'stress-strain':ti,ab,kw  )  NOT  (  'systematic review':ti  OR 'meta-analysis':ti  OR 'review article':ti  OR 'survey paper':ti  OR protocol:ti  ) | 89 |
| **Web of Science** | TS=(  (  stress OR "mental stress" OR "psychological stress" OR "perceived stress"  OR "emotional stress" OR "subjective stress" OR "acute stress"  OR "cognitive stress" OR "psychosocial stress"  OR "stress level*" OR "stress state*"  )  AND  (  detect* OR recogni* OR classif* OR predict* OR monitor* OR identif*  OR assess* OR estimat* OR discriminat*  )  AND  (  noncontact OR "non-contact" OR "contact-free" OR contactless  OR unobtrusive OR unobtrus*  OR "camera-based" OR "video-based" OR "vision-based" OR webcam  OR "facial video" OR "RGB camera*" OR "video camera*"  OR "infrared camera*" OR "thermal camera*" OR "thermal imaging"  OR "thermal image*" OR thermography  OR radar OR "Doppler radar" OR FMCW OR "CW radar"  OR "millimeter wave" OR mmWave OR microwave  OR UWB OR "ultra-wideband"  OR WiFi OR "WiFi CSI" OR "wireless sensing" OR RFID  OR rPPG OR iPPG  OR "remote photoplethysmograph*"  OR "imaging photoplethysmograph*"  OR "bed sensor*" OR "smart bed*" OR mattress OR "bed-based"  OR piezoelectric OR "pressure sensor*"  OR "smart chair*" OR "seat sensor*" OR "chair sensor*"  OR "seat cushion*" OR "load cell*"  )  AND  (  physiolog* OR biosignal* OR biopotential* OR "vital sign*"  OR autonomic OR cardiorespiratory  OR PPG OR photoplethysmogra*  OR rPPG OR iPPG  OR "remote photoplethysmograph*"  OR "imaging photoplethysmograph*"  OR BVP OR "blood volume pulse"  OR BCG OR ballistocardiogra*  OR "heart rate"  OR HRV OR "heart rate variability"  OR PRV OR "pulse rate variability"  OR IBI OR "interbeat interval*"  OR respiration OR respiratory OR "respiratory rate"  OR "skin temperature" OR "body temperature"  OR SpO2 OR "oxygen saturation"  OR PWA OR "pulse wave amplitude"  )  AND  (  "artificial intelligence" OR "machine learning" OR "deep learning"  OR "supervised learning" OR "ensemble learning"  OR "pattern recognition" OR classifier*  OR "classification model*" OR "predictive model*"  OR "support vector machine*" OR SVM  OR "random forest*" OR "decision tree*"  OR "k-nearest neighbor*" OR KNN  OR "artificial neural network*"  OR "neural network*"  OR "convolutional neural network*"  OR "recurrent neural network*"  OR "long short-term memory" OR LSTM  OR CNN OR XGBoost OR AdaBoost  OR "logistic regression"  OR "naive bayes"  OR "linear discriminant analysis"  OR LDA  )  NOT  (  "oxidative stress" OR "heat stress" OR "thermal stress"  OR "mechanical stress" OR "cell stress"  OR "plant stress" OR "crop stress" OR "leaf stress"  OR "drought stress" OR "salt stress" OR "water stress"  OR "stress-strain"  )  NOT  (  plant* OR leaf OR "systematic review" OR "meta-analysis"  OR "review article" OR "survey paper" OR protocol  )  ) | 253 |
| **APA PsycINFO** | (  exp Psychological Stress/  or stress.ti,ab,id.  or "mental stress".ti,ab,id.  or "psychological stress".ti,ab,id.  or "perceived stress".ti,ab,id.  or "emotional stress".ti,ab,id.  or "subjective stress".ti,ab,id.  or "acute stress".ti,ab,id.  or "cognitive stress".ti,ab,id.  or "psychosocial stress".ti,ab,id.  or "stress level*".ti,ab,id.  or "stress state*".ti,ab,id.  )  and  (  detect*.ti,ab,id.  or recogni*.ti,ab,id.  or classif*.ti,ab,id.  or predict*.ti,ab,id.  or monitor*.ti,ab,id.  or identif*.ti,ab,id.  or assess*.ti,ab,id.  or estimat*.ti,ab,id.  or discriminat*.ti,ab,id.  )  and  (  noncontact.ti,ab,id.  or "non-contact".ti,ab,id.  or contactless.ti,ab,id.  or unobtrusive.ti,ab,id.  or unobtrus*.ti,ab,id.  or "camera-based".ti,ab,id.  or "video-based".ti,ab,id.  or webcam.ti,ab,id.  or "facial video".ti,ab,id.  or "infrared camera*".ti,ab,id.  or "thermal camera*".ti,ab,id.  or "thermal imaging".ti,ab,id.  or thermography.ti,ab,id.  or radar.ti,ab,id.  or "doppler radar".ti,ab,id.  or FMCW.ti,ab,id.  or "CW radar".ti,ab,id.  or "millimeter wave".ti,ab,id.  or mmWave.ti,ab,id.  or microwave.ti,ab,id.  or UWB.ti,ab,id.  or "ultra-wideband".ti,ab,id.  or WiFi.ti,ab,id.  or "WiFi CSI".ti,ab,id.  or "wireless sensing".ti,ab,id.  or RFID.ti,ab,id.  or rPPG.ti,ab,id.  or iPPG.ti,ab,id.  or "remote photoplethysmograph*".ti,ab,id.  or "imaging photoplethysmograph*".ti,ab,id.  or "bed sensor*".ti,ab,id.  or "smart bed*".ti,ab,id.  or mattress.ti,ab,id.  or "bed-based".ti,ab,id.  or piezoelectric.ti,ab,id.  or "pressure sensor*".ti,ab,id.  or "smart chair*".ti,ab,id.  or "seat sensor*".ti,ab,id.  or "chair sensor*".ti,ab,id.  or "seat cushion*".ti,ab,id.  or "load cell*".ti,ab,id.  )  and  (  physiolog*.ti,ab,id.  or biosignal*.ti,ab,id.  or biopotential*.ti,ab,id.  or "vital sign*".ti,ab,id.  or autonomic.ti,ab,id.  or cardiorespiratory.ti,ab,id.  or PPG.ti,ab,id.  or photoplethysmogra*.ti,ab,id.  or rPPG.ti,ab,id.  or iPPG.ti,ab,id.  or "remote photoplethysmograph*".ti,ab,id.  or "imaging photoplethysmograph*".ti,ab,id.  or BVP.ti,ab,id.  or "blood volume pulse".ti,ab,id.  or BCG.ti,ab,id.  or ballistocardiogra*.ti,ab,id.  or "heart rate".ti,ab,id.  or HRV.ti,ab,id.  or "heart rate variability".ti,ab,id.  or PRV.ti,ab,id.  or "pulse rate variability".ti,ab,id.  or IBI.ti,ab,id.  or "interbeat interval*".ti,ab,id.  or respiration.ti,ab,id.  or respiratory.ti,ab,id.  or "respiratory rate".ti,ab,id.  or "skin temperature".ti,ab,id.  or "body temperature".ti,ab,id.  or SpO2.ti,ab,id.  or "oxygen saturation".ti,ab,id.  or PWA.ti,ab,id.  or "pulse wave amplitude".ti,ab,id.  )  and  (  exp Artificial Intelligence/  or exp Machine Learning/  or exp Deep Learning/  or "artificial intelligence".ti,ab,id.  or "machine learning".ti,ab,id.  or "deep learning".ti,ab,id.  or "supervised learning".ti,ab,id.  or "ensemble learning".ti,ab,id.  or "pattern recognition".ti,ab,id.  or classifier*.ti,ab,id.  or "classification model*".ti,ab,id.  or "predictive model*".ti,ab,id.  or "support vector machine*".ti,ab,id.  or SVM.ti,ab,id.  or "random forest*".ti,ab,id.  or "decision tree*".ti,ab,id.  or "k-nearest neighbor*".ti,ab,id.  or KNN.ti,ab,id.  or "artificial neural network*".ti,ab,id.  or "neural network*".ti,ab,id.  or "convolutional neural network*".ti,ab,id.  or "recurrent neural network*".ti,ab,id.  or "long short-term memory".ti,ab,id.  or LSTM.ti,ab,id.  or CNN.ti,ab,id.  or XGBoost.ti,ab,id.  or AdaBoost.ti,ab,id.  or "logistic regression".ti,ab,id.  or "naive bayes".ti,ab,id.  or "linear discriminant analysis".ti,ab,id.  or LDA.ti,ab,id.  )  not  (  "oxidative stress".ti,ab,id.  or "heat stress".ti,ab,id.  or "thermal stress".ti,ab,id.  or "mechanical stress".ti,ab,id.  or "cell stress".ti,ab,id.  or "plant stress".ti,ab,id.  or "crop stress".ti,ab,id.  or "leaf stress".ti,ab,id.  or "drought stress".ti,ab,id.  or "salt stress".ti,ab,id.  or "water stress".ti,ab,id.  or "stress-strain".ti,ab,id.  )  not  (  "systematic review".ti.  or "meta-analysis".ti.  or "review article".ti.  or "survey paper".ti.  or protocol.ti.  ) | 0 |
